# Assessing the causal associations of different types of statins use and knee/hip osteoarthritis: A Mendelian randomization study

**DOI:** 10.1371/journal.pone.0297766

**Published:** 2024-04-22

**Authors:** Xin Chen, Xin Huang, Youqun Liu, Zhiwei Zhang, Jiliang Chen

**Affiliations:** 1 Department of Urology, Mindong Hospital Affiliated to Fujian Medical University, Fuan, Fujian Province, People’s Republic of China; 2 Department of Orthopedics, Mindong Hospital Affiliated to Fujian Medical University, Fuan, Fujian Province, People’s Republic of China; 3 Department of Nursing, Xiangan Hospital of Xiamen University, School of Medicine, Xiamen University, Xiamen, People’s Republic of China; Catholic University of Brasilia, BRAZIL

## Abstract

**Objective:**

This study comprehensively evaluated the causal relationship between different types of statins use and knee/hip osteoarthritis (OA) using a two-sample and multivariate Mendelian randomization (MR) method.

**Methods:**

MR analysis was conducted using publicly available summary statistics data from genome-wide association studies (GWAS) to assess the causal associations between total statins use (including specific types) and knee/hip OA. The primary analysis utilized the inverse variance-weighted (IVW) method, with sensitivity analysis conducted to assess robustness. Multivariable MR (MVMR) analysis adjusted for low-density lipoprotein cholesterol (LDL-C), intermediate-density lipoprotein cholesterol (IDL-C), high-density lipoprotein cholesterol (HDL-C), and body mass index (BMI).

**Results:**

The MR analysis revealed a significant inverse association between genetically predicted total statins use and the risk of knee OA (OR = 0.950, 95%CI: 0.920–0.982, p = 0.002) as well as hip OA (OR = 0.932, 95%CI: 0.899–0.966, p <0.001). Furthermore, this study highlighted a reduced risk of knee/hip OA with the use of atorvastatin and simvastatin. Rosuvastatin use was associated with a decreased risk of hip OA but showed no association with knee OA. MVMR results indicated no correlation between exposure factors and outcomes after adjusting for LDL-C or IDL-C. HDL-C may not significantly contribute to statin-induced osteoarthritis, while BMI may play an important role.

**Conclusion:**

This study provides compelling evidence of the close relationship between statin use and a reduced risk of knee/hip OA, particularly with atorvastatin and simvastatin. LDL-C and IDL-C may mediate these effects. These findings have important implications for the clinical prevention and treatment of knee/hip OA.

## Introduction

Osteoarthritis (OA) is a chronic and debilitating disease that poses significant challenges to human well-being and imposes a substantial burden on society’s healthcare systems. Characterized by symptoms such as pain, morning stiffness, and joint creaking, OA leads to joint instability, physical limitations, and a decreased quality of life [1]. Due to the growing prevalence of obesity and an aging population, the global incidence of OA continues to escalate [2], with an estimated 240 million individuals affected worldwide [3]. Among the various joints in the body, the knee and hip are particularly prone to this degenerative condition [2]. While symptomatic treatments exist to alleviate pain and enhance joint mobility, effective conservative therapies capable of halting disease progression remain elusive [4]. Consequently, understanding the underlying factors contributing to OA pathogenesis is crucial for prevention and amelioration of this debilitating condition.

Statins, recognized as lipid-lowering agents that inhibit 3-Hydroxy-3-methylglutaryl coenzyme A reductase, are widely prescribed for cholesterol management in adults and for the prevention of atherosclerotic cardiovascular disease in both primary and secondary settings [5]. Atorvastatin ranks as the most commonly utilized statin, followed by simvastatin and rosuvastatin [6]. In recent years, researchers have focused on the effects of statins on bone and joint health, with several articles examining whether these medications may potentially promote OA. In vitro and animal experiments have demonstrated that atorvastatin can reduce glycosaminoglycan release [7], mitigate monosodium iodoacetate-induced effects through the STAT1-caspase-3 signaling pathway [8], enhance antioxidant enzyme expression [9], suppress oxidation reactions, and inhibit mitochondrial apoptosis [10]. However, cohort studies and meta-analyses have produced contradictory findings regarding the link between statins and the progression of osteoarthritis (OA). Interestingly, studies have suggested disparate effects of atorvastatin and rosuvastatin on osteoarticular inflammation [11], with rosuvastatin potentially exacerbating pain in patients with OA [12]. The conflicting results, combined with inherent limitations in observational studies such as residual confounding and reverse causality, hinder a conclusive evaluation of the causal connection between statin usage and knee/hip osteoarthritis (OA).

To address these biases associated with conventional observational studies, we employed a novel approach utilizing Mendelian randomization (MR) to explore the causal influence of statins on OA. MR employs genetic variations, specifically single nucleotide polymorphisms (SNPs), as instrumental variables (IVs) to serve as proxies for both exposure (statin use) and outcome (knee/hip OA) [13]. By utilizing the random allocation of genetic variants during conception, Mendelian randomization (MR) inherently mitigates confounding factors and avoids the impact of reverse causation. In this study, we employed a two-sample MR analysis to evaluate the causal impact of total statins usage as well as three commonly prescribed statins on the occurrence of knee OA and hip OA. Furthermore, to shed light on potential mechanistic pathways, we conducted multivariate MR (MVMR) analyses to evaluate the mediating effects of low-density lipoprotein cholesterol (LDL-C), intermediate-density lipoprotein cholesterol (IDL-C), high-density lipoprotein cholesterol (HDL-C) and body mass index (BMI).

## Materials and methods

### Study design

**Fig 1:**
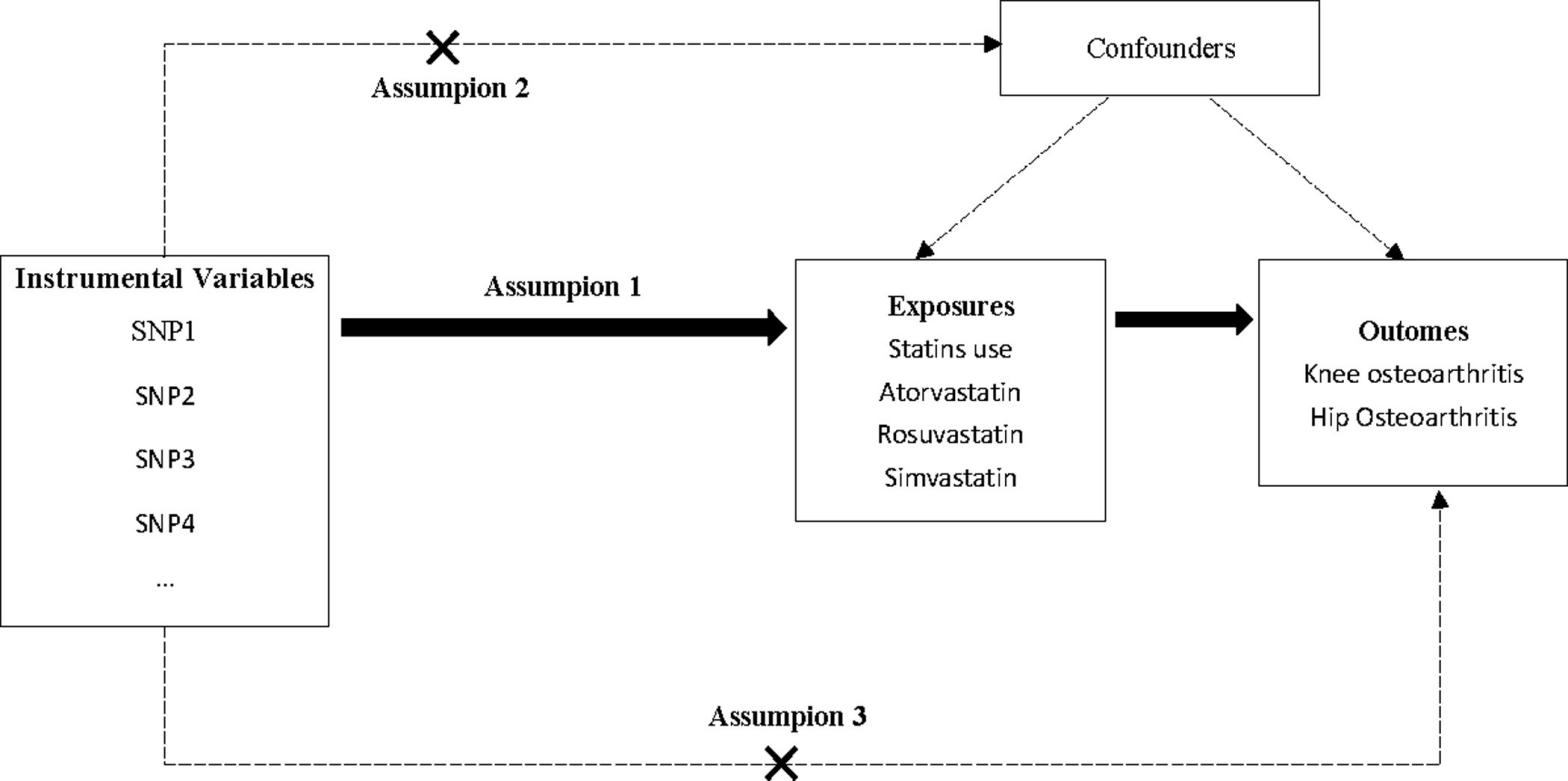
Diagram of Mendelian randomization analysis. Arrows represent associations. Assumption 1: the IVs correlate strongly with exposures. Assumption 2: the IVs are not associated with confounders. Assumption 3: the IVs influence outcomes only through exposures.

Since our analysis involved the reanalysis of publicly available data, there was no need for additional ethical approval. In this study, we employed a MR approach, utilizing genetic variations, particularly SNPs, as IVs to examine potential causal relationships between statin exposure and OA. To ensure the robustness of our MR analysis, the selected IVs needed to fulfill three key assumptions [14]: (1) a strong association between the IVs and the exposure; (2) no significant correlation between the IVs and confounding factors; and (3) the IVs impacts the risk of the outcome solely through the exposure, without any alternative pathways. We utilized a two-sample MR analysis to examine the impact of total statins use as well as three frequently prescribed statins (atorvastatin, rosuvastatin, and simvastatin) on knee/hip OA. For statins that demonstrate a causal relationship with knee/hip OA, we performed a MVMR analysis to evaluate the standalone impact of statin use on knee/hip OA and investigate potential mediators that may explain the association between exposure and outcome. The study design is depicted in Fig 1.

### Data sources

#### Statin use data

The information concerning statin use and OA in our study was acquired from the IEU Open GWAS database, which is accessible through the GWAS catalogue. We focused on analyzing both total statins use and three commonly prescribed statins. The data on total statins use were obtained by extracting pooled statistics from the Genome-wide Association Study (GWAS) conducted in the 5th version of the FinnGen study (https://finngen.gitbook.io/documentation/). This dataset consisted of a remarkable cohort of 218,792 participants of European ancestry, encompassing 68,782 cases and 150,010 controls. Additionally, we incorporated three common statins use as exposures: atorvastatin use (13,851 cases and 449,082 controls), rosuvastatin use (2,870 cases and 460,063 controls), and simvastatin use (52,427 cases and 410,506 controls) [15]. The dataset encompassed over 9.85 million SNPs and included individuals of European ancestry, both males and females, sourced exclusively from the same study.

#### OA data

Our investigation into OA involved the analysis of two common subtypes: knee OA and hip OA. To acquire the relevant data, we accessed publicly available GWAS databases. For knee OA, we examined a cohort comprising 24,955 cases and 378,169 controls [16], while for hip OA, the dataset consisted of 15,704 cases and 378,169 controls [16]. The corresponding GWAS IDs for knee OA and hip OA are ebi-a-GCST007090 and ebi-a-GCST007091, respectively. These datasets were obtained from the same study, which involved a comprehensive analysis of nearly 30 million SNPs. Furthermore, we obtained GWAS data pertaining to the potential pathogenesis of statins and OA, including LDL-C, IDL-C and HDL-C data investigated by Johannes Kettunen et al. [17], as well as BMI data sourced from the Genetic Investigation of ANthropometric Traits database [18]. All individuals included in these datasets are of European ancestry exclusively, and to our knowledge, the exposure (statin use) investigated in this study, as well as the datasets involved in the MVMR analysis (LDL-C, IDL-C, HDL-C and BMI), do not overlap with the GWAS on outcome (knee/hip OA). All detailed descriptions are included in Table 1.

**Table 1:** Details of datasets included in analyses.

	Trait	Sample size (Case/Control)	Population	Consortium	Reference	GWAS-ID	Year
Exposures	Statins use	68,782/ 150,010	European	Finngen	https://www.finngen.fi/en	finn-b-RX_STATIN	2021
	Atorvastatin use	13,851/449,082	European	MRC-IEU	Gibran Hemani et al.	ukb-b-10008	2018
	Rosuvastatin use	2,870/460,063	European	MRC-IEU	Gibran Hemani et al.	ukb-b-13664	2018
	Simvastatin use	52,427/ 410,506	European	MRC-IEU	Gibran Hemani et al.	ukb-b-11268	2018
Outcome	Knee osteoarthritis	24,955/ 378,169	European	EBI	Ioanna Tachmazidou et al.	ebi-a-GCST007090	2019
	Hip osteoarthritis	15,704/ 378,169	European	EBI	Ioanna Tachmazidou et al.	ebi-a-GCST007091	2019
MVMR	LDL-C	21,559	European	NA	Johannes Kettunen et al.	met-c-895	2016
	IDL-C	19,273	European	NA	Johannes Kettunen et al.	met-c-867	2016
	HDL-C	21,555	European	NA	Johannes Kettunen et al.	met-c-864	2016
	BMI	681,275	European	GIANT	Loic Yengo et al.	ieu-b-40	2018

Abbreviation: MRC-IEU: Medical Research Council Integrative Epidemiology Unit; EBI: European Bioinformatics Institute; NA: Not Applicable; GIANT: Genetic Investigation of ANthropometric Traits; LDL-C: Low density lipoprotein cholesterol; IDL-C: Intermediate density lipoprotein cholesterol; HDL-C: High density lipoprotein cholesterol; BMI: Body Mass Index.

### Instrument selection

The selection of IVs in our study adhered to the fundamental assumptions of MR analysis, as outlined previously. To identify SNPs that exhibited significant associations with statin use (p < 5 × 10–8), we leveraged the GWAS pooled data. To ensure the independence of the chosen SNPs, we employed a clustering approach with a window size of 10,000 kb while maintaining low levels of linkage disequilibrium (R2 < 0.001) [19]. Furthermore, we calculated the F-statistic—a metric evaluating the strength of each SNP—using the formula F = beta2/se2. Here, beta represents the SNP’s effect size, indicating the magnitude of its impact, while se denotes the standard error associated with the SNP.

To ensure robustness in our analysis, we excluded instruments exhibiting weak associations by removing those with F-statistics below 10 [20]. Moreover, we implemented stringent filtering procedures to eliminate palindromic or incompatible SNPs. Subsequently, we employed the MR pleiotropic residual sum and outlier (MR-PRESSO) test to detect any outlier SNPs that may have demonstrated pleiotropic effects [21]. Any identified outliers were subsequently discarded. The remaining set of SNPs served as the IVs for subsequent MR testing.

### Statistical analysis

In this study, we conducted a comprehensive investigation into the causal association between genetic predictions, statin use, and the risk of OA. Our primary analysis employed a random-effects inverse variance weighting (IVW) approach [22], complemented by the inclusion of MR Egger and weighted median methods for additional verification, thereby ensuring the robustness of our effect estimates derived from the IVW method [23, 24]. To evaluate potential biases in the observed causality, we performed sensitivity analyses and employed Cochran’s Q test to examine heterogeneity [25]. The heterogeneity was further visualized through funnel plots.

To address concerns regarding horizontal pleiotropy, we utilized MR-Egger’s intercept term and conducted the MR-PRESSO global test as additional measures [23]. By leveraging MR-PRESSO, we were able to identify outliers in the associations and re-evaluate the causal relationship after excluding these outliers [21]. Additionally, we employed leave-one-out analysis in data visualization to detect potential outliers that exerted significant influence on the aggregated IVW estimates [26]. Forest plots were employed to assess the effect estimates associated with genetic variants and their relationship with statin use, while scatterplots allowed us to explore relationships, detect outliers, and uncover trends.

Expanding upon the traditional MR approach, we incorporated MVMR, which accounts for potential confounding factors similar to multivariate regression [27]. Considering that there are genetically associated factors with statin use, such as LDL-C, IDL-C, HDL-C, and BMI, which are strongly associated with OA [28–30], we postulated that these factors may potentially act as mediators in the association between statin use and the reduced risk of OA. Therefore, if statin use was found to be causally linked to knee/hip OA in the univariate MR analysis, we further performed MVMR analysis to investigate the impact of these three mediators on OA. The causal effect estimation was performed using the IVW method.

The association between statin use and the risk of OA disease was quantified using odds ratios (OR) accompanied by their corresponding 95% confidence intervals (CI). All statistical analyses were performed using R programming language (version 4.1.2) with the utilization of "TwoSampleMR," "MR-PRESSO," and "MVMR" packages (version 0.3). Moreover, within the MR analyses, we assessed the impact of both total statins use and three common statins use on knee/hip OA. To determine statistical significance, we utilized the Bonferroni-corrected p-value (p < 0.05/8 = 6.25 × 10^−3^) as the threshold. An association was considered suggestive when the P value fell within the range of 6.25 × 10^−3^ and 0.05.

## Result

After meticulous exclusion of SNPs that failed to meet rigorous quality control criteria (p < 5×10−8, R2 < 0.001, F > 10), and employing the MR-PRESSO method to eliminate any potential outliers, our investigation into knee OA yielded a set of 55 SNPs associated with total statins use, 17 SNPs linked to atorvastatin use, 5 SNPs associated with rosuvastatin use, and 30 SNPs correlated with simvastatin use, serving as IVs. In parallel, for hip OA research, we identified 56 SNPs as IVs for total statins use, 18 SNPs for atorvastatin use, 5 SNPs for rosuvastatin use, and 32 SNPs for simvastatin use. Impressively, the computed F statistics revealed a robust correlation between the IVs and exposure, with all F values surpassing the threshold of 10. The detailed information regarding the IVs used in our MR analysis is available in the S1-S8 Tables in S1 File.

### Causal effects of statins use and three common statins use on knee OA

**Fig 2:**
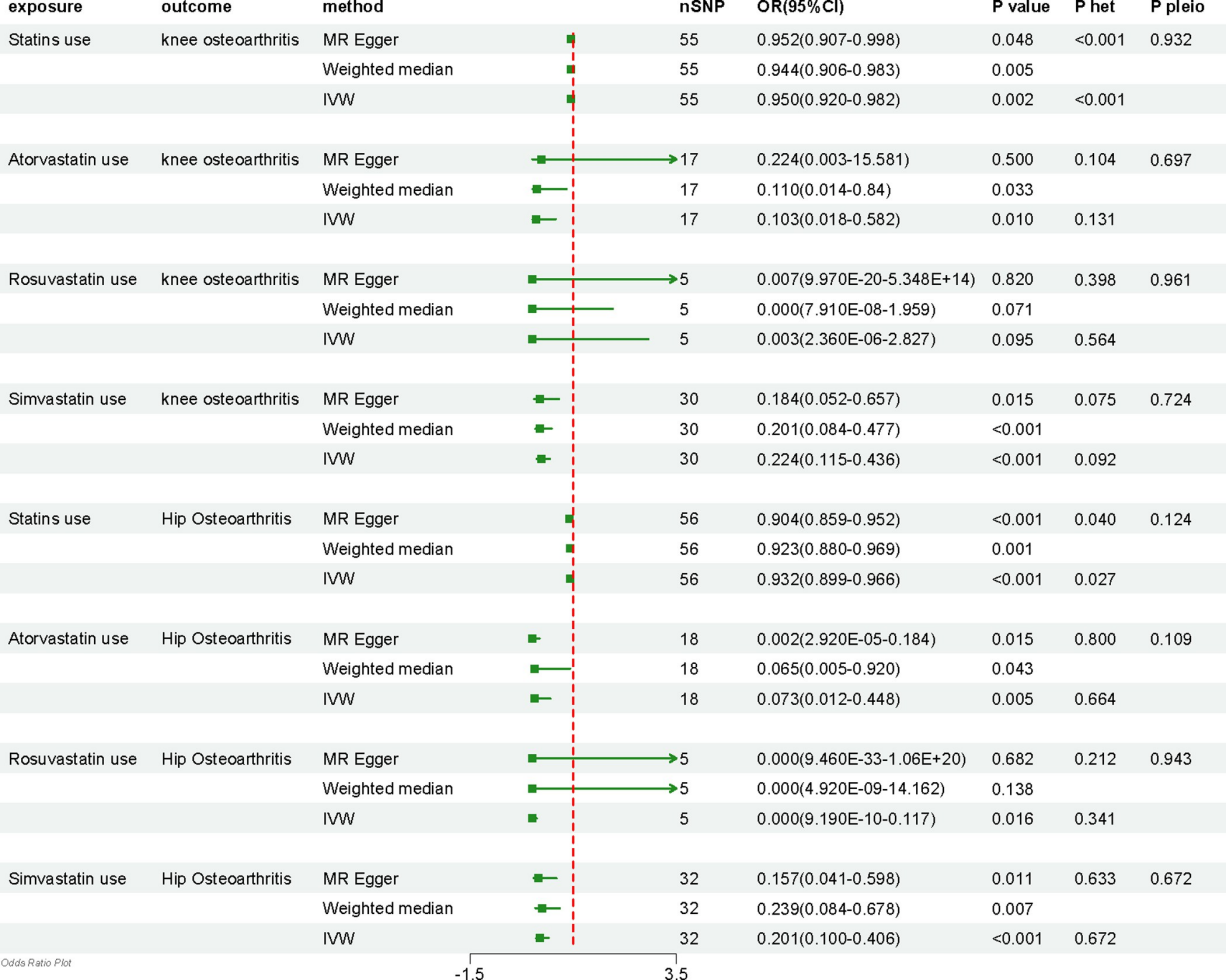
The potential causal relationships between different types of statins use and knee/hip osteoarthritis were examined using various MR methods, including MR Egger, weighted median, inverse variance weighted.

The investigation into the causal effects of statins and their individual types on knee OA revealed intriguing findings. Notably, a higher genetic predisposition towards total statins use and simvastatin use was linked to a decreased risk of Knee OA (statins use-Knee OA: OR = 0.950, 95% CI = 0.920–0.982, p = 0.002; simvastatin use-Knee OA: OR = 0.224, 95% CI = 0.115–0.436, p < 0.001) (Fig 2). Higher genetic predisposition to atorvastatin use was suggestively associated with a reduced risk of knee OA (atorvastatin use-knee OA: OR = 0.103, 95% CI = 0.018–0.582, p = 0.010) (Fig 2). Surprisingly, no discernible causal relationship between rosuvastatin use and susceptibility to Knee OA was observed during this study (rosuvastatin use-Knee OA: OR = 0.003, 95% CI = 0.000–2.827, p = 0.095) (Fig 2). Encouragingly, the findings derived from MR-Egger and weighted median analyses exhibited substantial concordance with the IVW approach in terms of their directional consistency (Fig 2).

In order to detect potential heterogeneity in the effect of statins use and three common statins use on Knee OA, sensitivity analyses employing MR-Egger regression and IVW-based analysis were conducted. Remarkably, significant heterogeneity was only detected in relation to statins use (MR-Egger: p < 0.001; IVW: p < 0.001) (Fig 2); however, this observed heterogeneity was deemed acceptable as the study adopted a random-effects IVW as the primary outcome [31]. Conversely, no substantial heterogeneity was identified among the other three types of statins (all p > 0.05), as depicted in the funnel plot presented in Supplementary material figure. Furthermore, the MR-Egger intercept test results yielded no evidence of pleiotropy at any level (all p > 0.05). To avoid reliance on a single IV, ensuring the robustness of the causal associations, a comprehensive leave-one-out analysis was performed, systematically excluding each SNP. Encouragingly, the final results confirmed the robustness of the MR analysis. Scatter plots and forest plots depicting the causal effects also exhibited no conspicuous heterogeneity among the statin-related SNPs (S1-S16 Figs in S1 File).

### Causal effects of statins use and three common statins use on hip OA

In the context of hip OA, our investigation uncovered noteworthy insights into the causal effects of statins and their individual types. Strikingly, a heightened genetic predisposition towards statins use, atorvastatin use and simvastatin use was link to a reduced risk of HOA (statins use-Hip OA: OR = 0.932, 95% CI = 0.899–0.966, p < 0.001; atorvastatin use-Hip OA: OR = 0.073, 95% CI = 0.012–0.448, p = 0.012–0.448; simvastatin use-Hip OA: OR = 0.201, 95% CI = 0.100–0.406, p < 0.001) (Fig 2). Higher genetic predisposition to rosuvastatin use was suggestively associated with a reduced risk of knee OA (rosuvastatin use-Hip OA: OR = 0.000, 95% CI = 0.000–0.117, p = 0.016) (Fig 2). Importantly, the findings from MR-Egger and weighted median analyses were in substantial agreement with the IVW approach in terms of directional consistency (Fig 2).

To ascertain potential heterogeneity in the effect of statins use and three common statins use on HOA, we conducted sensitivity analyses employing MR-Egger regression and IVW-based analysis. As depicted in Fig 2, significant heterogeneity was solely observed in relation to statins use (MR-Egger: p = 0.040; IVW: p = 0.027). However, no substantial heterogeneity was identified among the other three types of statins use (all p > 0.05), as illustrated in the funnel plot provided in S1 File. Furthermore, the MR-Egger intercept test results provided no evidence of pleiotropy at any level (all p > 0.05). To further validate the causal associations, a comprehensive leave-one-out analysis was conducted, which supported the robustness of the MR analysis. Consistent effects across the statin-related SNPs were also observed in the scatter plots and forest plots (S17-S32 Figs in S1 File).

### MVMR of causal effects of statins use and three common statins use on knee/hip OA

In order to delve further into the causal effects of statins and their individual types on knee and hip OA, we conducted MVMR. Notably, upon adjusting for LDL-C or IDL-C, the gene-predicted effects of total statins and the three common statins on knee and hip OA exhibited significant attenuation, ultimately becoming statistically insignificant (Table 2). However, when adjusting for HDL, the independent effects of gene-predicted total statins and the three common statins on knee and hip OA persisted (Table 2). Similarly, after controlling for BMI, the independent effect of gene-predicted total statins on hip OA remained apparent, while the genetically predicted independent effects of total statins on knee OA and the three common statins on knee and hip OA became non-significant (Table 2).

**Table 2:** Causal effects of statins use and three common statins use on the risk of knee/hip osteoarthritis from MVMR.

	Effect on KOA		Effect on HOA	
	OR (95% CI)	P	OR (95% CI)	P
Statins use without adjustment	0.950(0.920–0.982)	0.002	0.932(0.899–0.966)	<0.001
Statins use adjusted for LDL-C	1.031(0.959–1.108)	0.410	0.984(0.910–1.065)	0.691
Statins use adjusted for IDL-C	0.997(0.931–1.067)	0.935	0.950(0.883–1.022)	0.168
Statins use adjusted for HDL-C	0.952(0.921–0.984)	0.004	0.933(0.903–0.964)	<0.001
Statins use adjusted for BMI	0.984(0.938–1.032)	0.499	0.944(0.894–0.996)	0.004
Atorvastatin use without adjustment	0.103(0.018–0.582)	0.010	0.073(0.012–0.448)	0.005
Atorvastatin use				
adjusted for LDL-C	0.966(0.877–1.063)	0.727	0.448(0.015–13.013)	0.640
Atorvastatin use adjusted for IDL-C	0.469(0.028–7.960)	0.600	0.406(0.012–13.229)	0.220
Atorvastatin use adjusted for HDL-C	0.196(0.045–0.836)	0.028	0.068(0.012–0.381)	0.002
Atorvastatin use adjusted for BMI	0.128(0.186–8.636)	0.808	0.193(0.019–1.935)	0.162
Rosuvastatin use without adjustment	0.003(0.000–2.827)	0.095	0.000(0.000–0.117)	0.016
Rosuvastatin use				
adjusted for LDL-C	−−−	−−−	1.682e-6(1.465e-16-19298.106)	0.261
Rosuvastatin use adjusted for IDL-C	−−−	−−−	2.233e-06(5.460e-16-9131.631)	0.249
Rosuvastatin use adjusted for HDL-C	−−−	−−−	1.049e-05 (1.771e-08- 0.006)	<0.001
Rosuvastatin use adjusted for BMI	−−−	−−−	120.925(0.121–1.209e+05)	0.174
Simvastatin use without adjustment	0.224(0.115–0.436)	<0.001	0.201(0.100–0.406)	<0.001
Simvastatin use adjusted for LDL-C	1.718(0.253–5.410)	0.839	1.643(0.303–8.881)	0.564
Simvastatin use adjusted for IDL-C	0.764(0.172–3.394)	0.723	0.967(0.167–5.610)	0.940
Simvastatin use adjusted for HDL-C	0.277(0.134–0.576)	<0.001	0.242(0.107–0.547)	<0.001
Simvastatin use adjusted for BMI	0.606(0.253–1.453)	0.261	0.335(0.122–0.921)	0.034

## Discussion

We conducted a pioneering two-sample MR study to investigate the influence of three distinct classes of statins on the incidence of knee and hip OA. To the best of our understanding, this pioneering MR study is the first to examine the causal association between distinct classes of statins and the development of both knee and hip OA.

Our research revealed a notable link between the use of statins and a decreased risk of both knee and hip OA when not considering the specific class of statins. Furthermore, when considering specific statin drug types, the use of atorvastatin and simvastatin demonstrated a reduction in the incidence of knee OA and hip OA. Furthermore, the utilization of rosuvastatin demonstrated a reduction in the frequency of hip OA but did not show any impact on the occurrence of knee OA. Multivariate analysis indicated that LDL-C and IDL-C may serve as important mediators mediating this effect, as there was no correlation between any exposure factor and outcome when LDL-C or IDL-C were excluded. HDL-C may not have a significant influence on the development of statin-induced osteoarthritis. Moreover, BMI could potentially play a vital role in this process.

Previous studies have already emphasized the positive impacts of statins on cartilage synthesis, metabolism, and the suppression of OA. Our study further supports the positive effects of statins in preventing OA. In particular, atorvastatin has been shown to protect against cartilage degeneration following IL-1β stimulation and elicit anti-inflammatory effects through the stimulation of the STAT1-caspase-3 signaling pathway [32]. Studies by Pathak et al. demonstrated that atorvastatin can reduce the inflammatory response induced by monosodium iodoacetate in a rat joint model [8].In their study, Jiancongchen et al. found that atorvastatin could suppress matrix degradation in rat nucleus pulposus cells induced by TNF-α [5]. This effect was achieved through the reduction of NLRP3 activity and NF-κB signaling, ultimately promoting autophagy. However, some cohort studies, controlled trials, and meta-analyses have indeed no connection between statin use and OA progression. For example, in a 4-year follow-up study led by Nicola Veronese et al., involving 4448 participants, no reduction in the incidence of symptomatic and imaging OA was found with statin use. However, they did find that long-term use of atorvastatin could help alleviate joint pain [12]. It is crucial to note that the subjects in these studies were individuals at high risk or already suffering from OA, limiting the generalizability to the normal population. According to a controlled trial conducted by Yuanyuan Wang et al. [33, 34], a two-year regimen of oral atorvastatin (40 mg daily) did not demonstrate a significant reduction in cartilage volume loss among individuals with symptomatic OA when compared to the placebo group. However, this trial had a small sample size and specifically targeted patients with knee OA who had symptoms for more than six months, suggesting that this dose of atorvastatin may not be suitable for knee OA treatment. A meta-analysis of 11 trials found that the use of statins may not be associated with a decreased risk of OA incidence and progression, regardless of the specific joint site involved [11]. However, it is important to note that the meta-analysis included a restricted number of trials, all of which were cohort and case-control studies. The inclusion of these study types may introduce biases such as selection bias, recall bias, and misclassification bias. Interestingly, a recent MR analysis suggested that the genetic proxy effect of statins in lowering LDL-C levels may increase the risk of knee OA but not hip OA [35]. This finding contradicts our research results, possibly due to disparities between genetic agent effects and real drug effects, as the former may have less involvement in the joint’s inflammatory responses and other mechanisms. By directly investigating the genes related to lipid-lowering drugs as exposure factors, we were able to avoid these discrepancies.

The results for simvastatin align with numerous previous studies. Jana Riegger et al. demonstrated that simvastatin and fluvastatin have the ability to reduce cell death and catabolism in human chondrocytes following trauma. [36].Kenya Terabe et al. demonstrated the importance of inhibiting essential protein prenylation processes to facilitate simvastatin’s beneficial impact on chondrocyte redifferentiation [37]. Additionally, Toshikazu Tanaka et al. demonstrated the potential of simvastatin-conjugated gelatin hydrogel when administered intra-articularly as a treatment for OA [38]. These collective findings suggest a protective effect of simvastatin on bone and articular cartilage, although further investigation is needed to elucidate the specific underlying mechanisms.

Currently, there is limited research on the mechanism by which rosuvastatin influences OA progression. However, certain observational studies have suggested that the inhibitory effect of rosuvastatin on OA may not be as significant compared to atorvastatin. In a longitudinal study conducted by Nicola Veronese, it was found that rosuvastatin was associated with an increased risk of exacerbating knee OA pain. [12]. The majority of the analyzed studies had follow-up periods ranging from one month to one year., with relatively short durations for detecting clinical effects. We speculate that this may be partly attributed to statins themselves causing skeletal muscle pain. A meta-analysis conducted by J. Wang et al. suggested that atorvastatin and rosuvastatin may have divergent effects on OA progression [11]. Our study found that rosuvastatin inhibited the progression of hip OA but had no significant impact on knee OA. This discrepancy may be related to the greater hereditary contribution to hip OA compared to knee OA [16]. Additional research is necessary to confirm the mechanism of action of rosuvastatin in OA.

In our MVMR, we identified an association between LDL-C, IDL-C, and OA induced by all the statins studied, underscoring the importance of LDL-C and IDL-C as a factor in the effects of statins on knee and hip OA. The mechanisms through which LDL and IDL promotes OA have been elucidated in previous experiments [39–42], suggesting that these mechanisms may underlie the observed changes. Furthermore, our study demonstrated that statin-induced OA is not mediated by HDL-C, despite previous suggestions of HDL’s potential impact on OA [28]. Regarding the intermediate mediating effect of BMI, we hypothesized that it might play a role in the alleviation of OA by statins. In individuals with high BMI, the load-bearing pressure on the hip and knee joints is greater than that in normal-weight individuals, leading to increased friction between the articular cartilage and facilitating the development of knee and hip OA. Earlier studies have also demonstrated a link between elevated BMI and the progression of arthritis [29, 30, 43]. However, even after accounting for BMI as an intermediate variable, the effects of total statins and simvastatin on hip OA remained significant. This suggests that BMI may have a limited impact on these two processes, highlighting the need for additional research to investigate the underlying reasons for these discrepancies.

Our study has certain limitations. First, it is crucial to recognize that MR studies cannot completely rule out horizontal pleiotropy and other potential direct causal pathways. Second, our analysis primarily relied on databases that consisted mainly of individuals with European ancestry, which may restrict the applicability of our findings to populations from other regions, especially developing countries. Third, Because of the nature of utilizing genetic variants linked to statin use, our study was unable to examine the distinct impacts of varying dosing concentrations and durations of administration on knee and hip OA outcomes. These factors may have considerable implications for the development and symptomatic progression of OA. Fourth, we did not explore specific variables through which statins affect the osteoarthritic process and the relative contributions of each variable.

In conclusion, our groundbreaking MR study offers valuable insights into the impact of various statins on the risk of knee and hip OA. The results indicate that both atorvastatin and simvastatin exhibit protective effects against both knee and hip OA. However, the outcomes associated with rosuvastatin demonstrate mixed findings depending on the specific joint site. LDL-C and IDL-C emerged as an essential mediator of the observed effects, highlighting its critical role in the relationship between statins and OA. Our findings add to the expanding body of evidence that supports the potential advantages of statins in preventing OA. Nonetheless, additional research is necessary to unravel the underlying mechanisms and address the limitations present in this study.

## Conclusion

In conclusion, our study identified a robust correlation between the use of statins and a decreased risk of knee and hip osteoarthritis, particularly with atorvastatin and simvastatin. LDL-C and IDL-C may play a role in mediating this effect, while BMI is also a contributing factor. HDL-C does not appear to have an impact on these outcomes. These findings provide valuable insights for further research.

## Supporting information

S1 File S1-S32 Figs and S1-S8 Tables are included in file. (DOCX)

## Abbreviations

OA: Osteoarthritis
MR: Mendelian randomization
SNPs: single nucleotide polymorphisms
IVs: instrumental variables
IDL: intermediate-density lipoprotein
HDL: high-density lipoprotein
BMI: body mass index
MVMR: multivariate mendelian randomization
GWAS: genome-wide association study
MR-PRESSO: MR-Pleiotropy Residual Sum and Outlier
IVW: inverse variance weighting
OR: odds ratios
CI: confidence intervals
LDL-C: low-density lipoprotein cholesterol
IDL-C: intermediate-density lipoprotein cholesterol
HDL-C: high-density lipoprotein cholesterol
BMI: body mass index
