# Supplementary material for: Assessing the causal associations of different types of statins use and knee/hip osteoarthritis: A Mendelian randomization study
Source: PLoS One. 2024 Apr 22;19(4):e0297766. doi: 10.1371/journal.pone.0297766 (PMC11034643; doi:10.1371/journal.pone.0297766)
Supplement: S1 File — (DOCX) [file pone.0297766.s001.docx]

**Assessing the causal associations of different types of statins use and knee/hip osteoarthritis: A Mendelian randomization study**

**Contents**

Figure S1-S4 the plots of the effect of statins use on knee osteoarthritis 1

Figure S5-S8 the plots of the effect of atorvastatin use on knee osteoarthritis 4

Figure S9-S12 the plots of the effect of rosuvastatin use on knee osteoarthritis 7

Figure S13-S16 the plots of the effect of simvastatin use on knee osteoarthritis 9

Figure S17-S20 the plots of the effect of statins use on hip osteoarthritis 12

Figure S21-S24 the plots of the effect of atorvastatin use on hip osteoarthritis 15

Figure S25-S28 the plots of the effect of rosuvastatin use on knee osteoarthritis 18

Figure S29-S32 the plots of the effect of simvastatin use on hip osteoarthritis 20

Table S1 Genome-wide significant SNPs associated with statins use in knee osteoarthritis 23

Table S2 Genome-wide significant SNPs associated with statins use in hip osteoarthritis 25

Table S3 Genome-wide significant SNPs associated with atorvastatin use in knee osteoarthritis 27

Table S4 Genome-wide significant SNPs associated with atorvastatin use in hip osteoarthritis 28

Table S5 Genome-wide significant SNPs associated with rosuvastatin use in knee osteoarthritis 29

Table S6 Genome-wide significant SNPs associated with rosuvastatin use in hip osteoarthritis 30

Table S7 Genome-wide significant SNPs associated with simvastatin use in knee osteoarthritis 31

Table S8 Genome-wide significant SNPs associated with simvastatin use in hip osteoarthritis 32


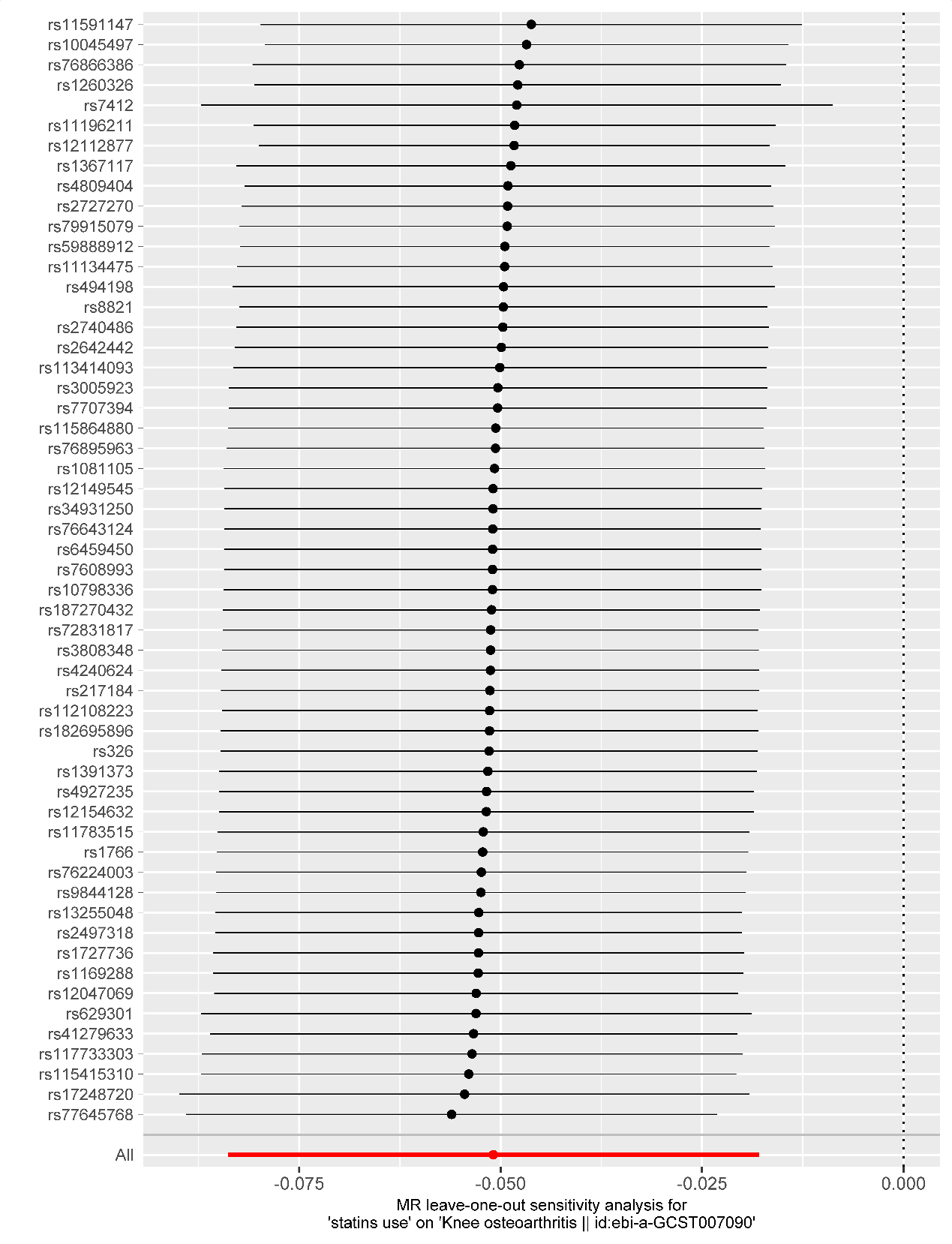


**Figure S1 the leave-one-out plot of the effect of statins use on knee osteoarthritis**


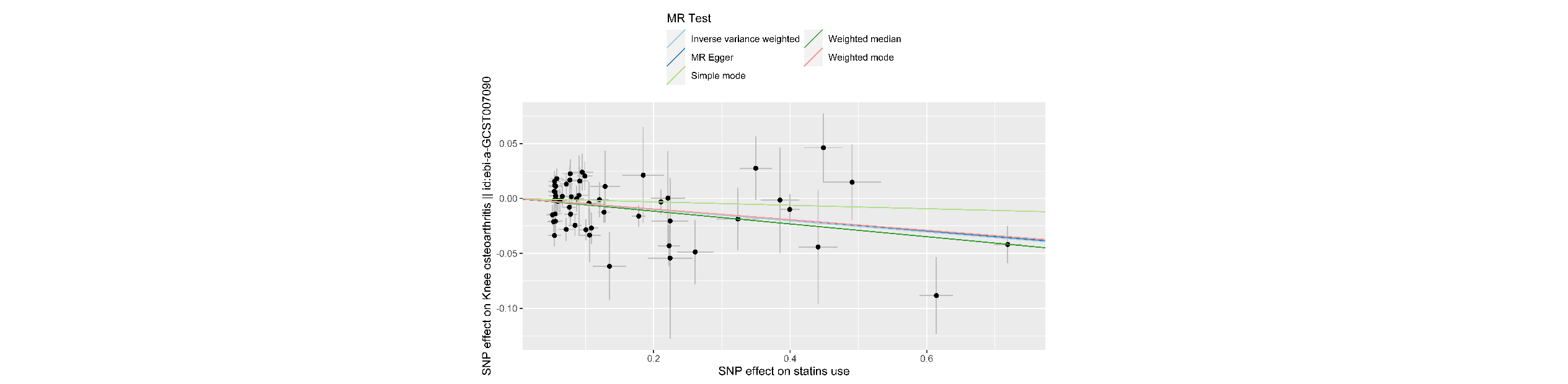


**Figure S2 the scatter plot of the effect of statins use on knee osteoarthritis**


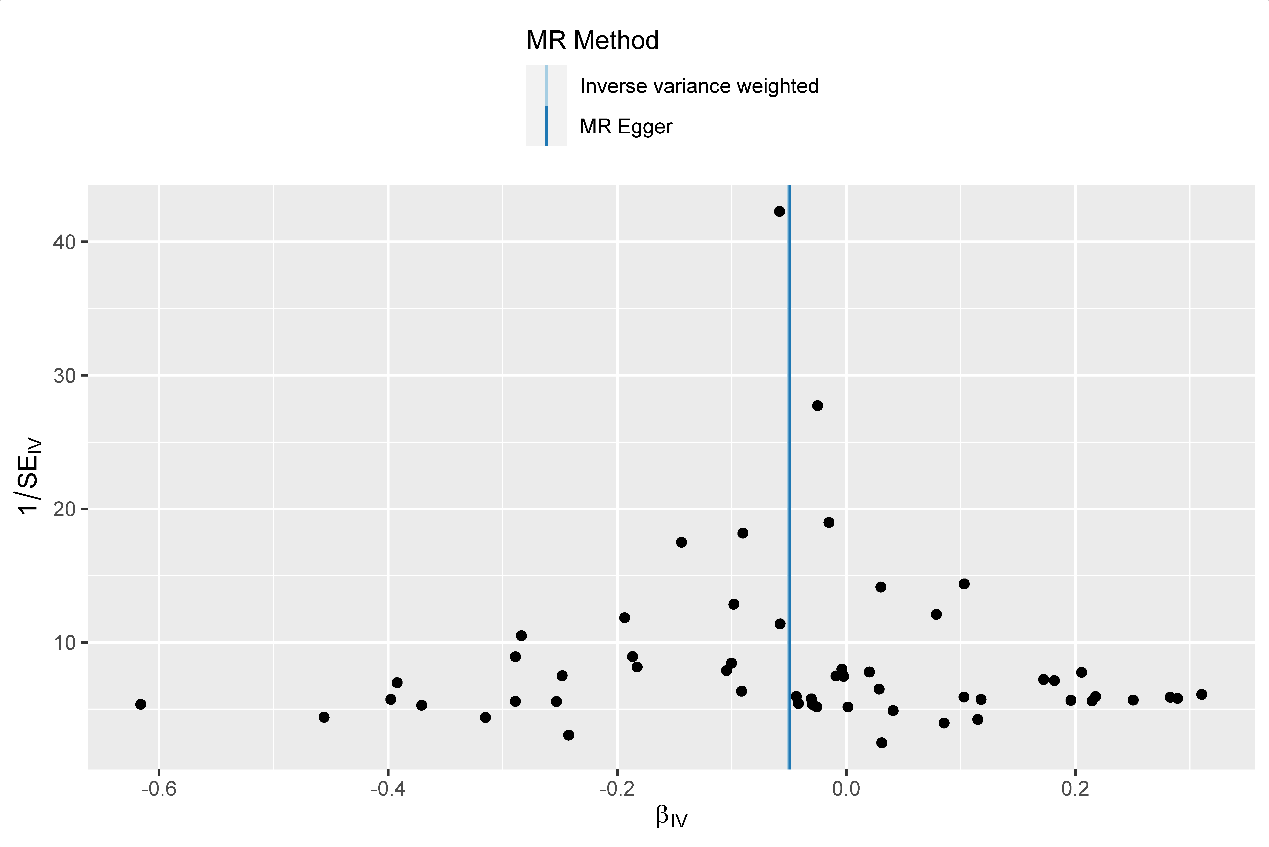


**Figure S3 the funnel plot of the effect of statins use on knee osteoarthritis**


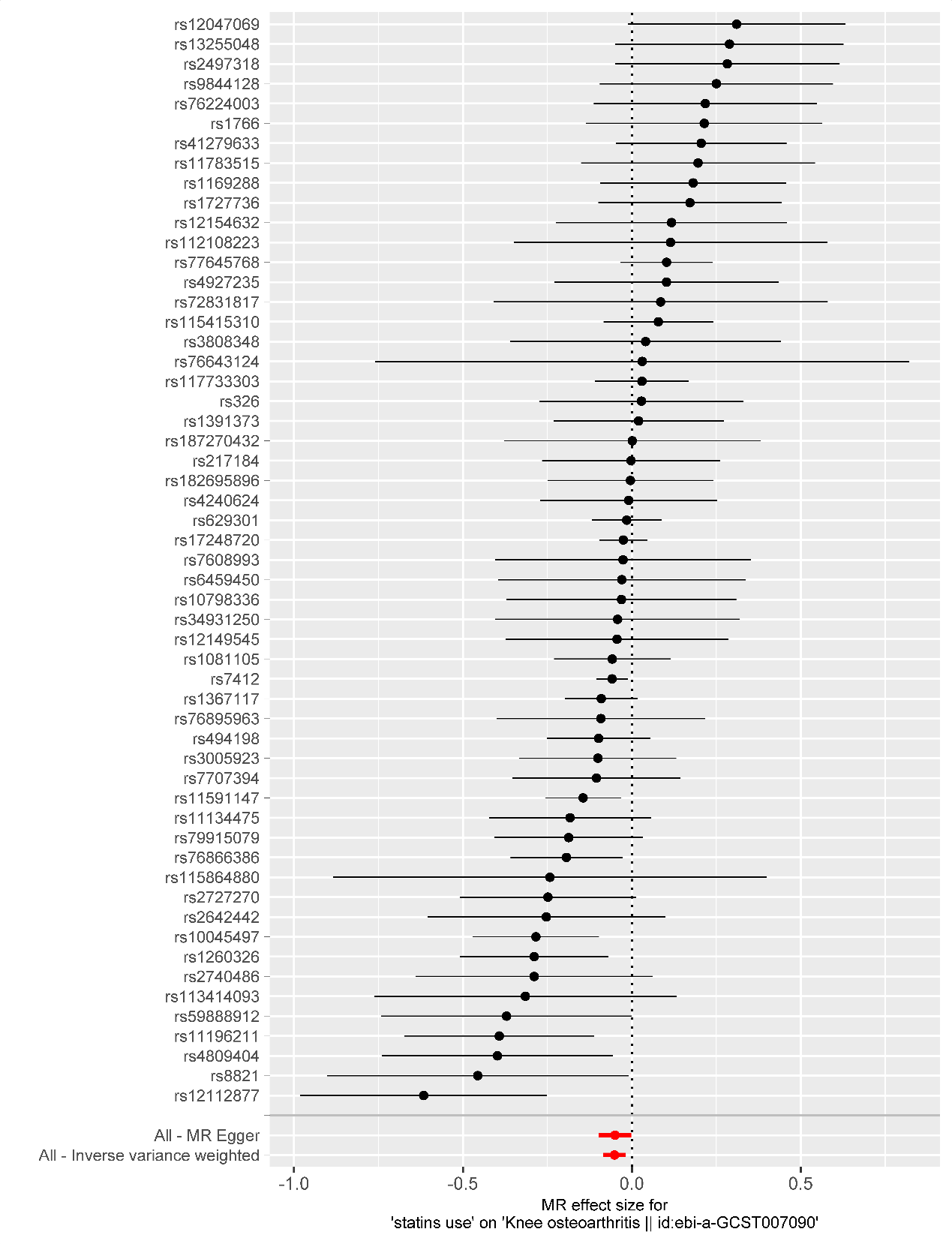


**Figure S4 the forest plot of the effect of statins use on knee osteoarthritis**


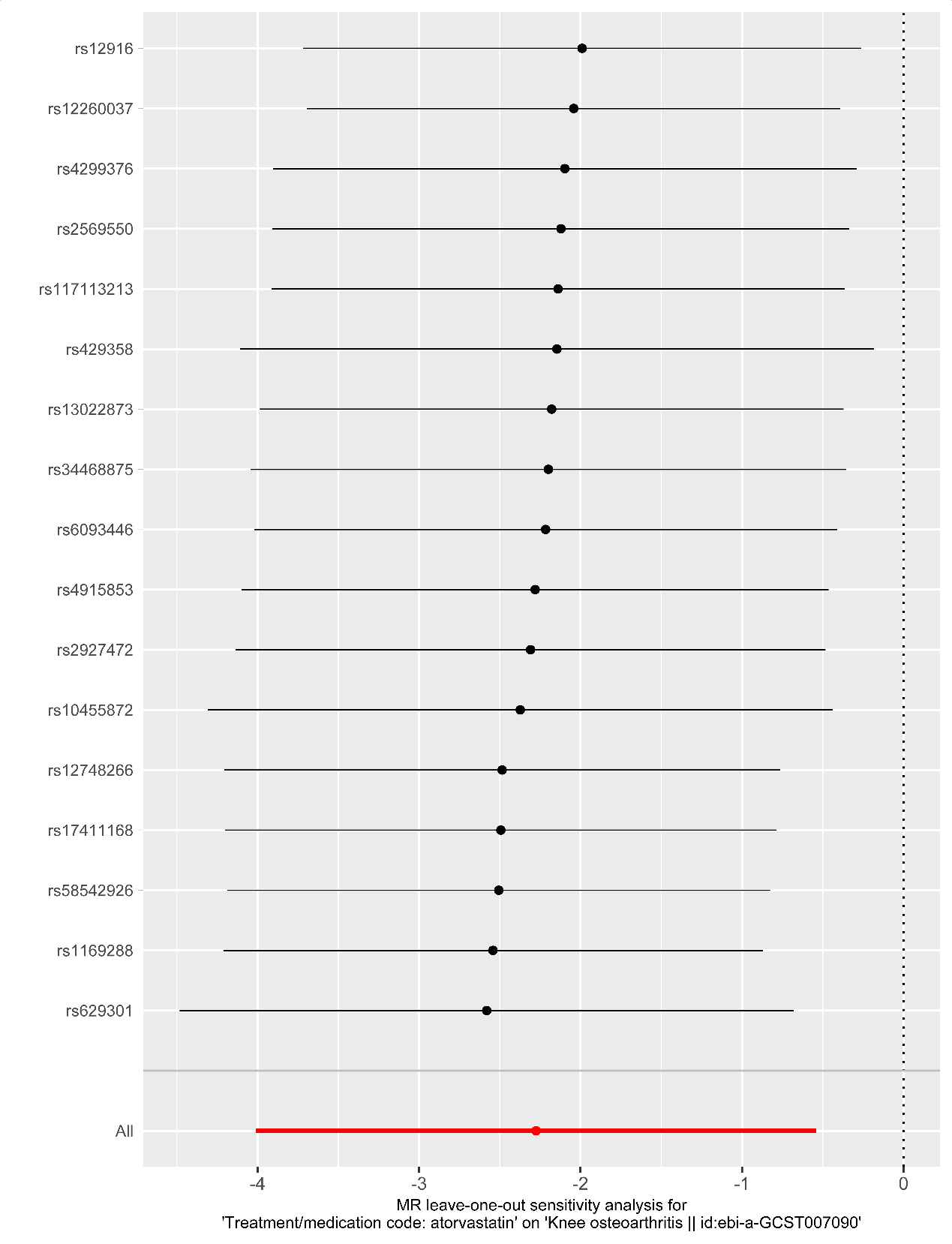


**Figure S5 the leave-one-out plot of the effect of atorvastatin use on knee osteoarthritis**


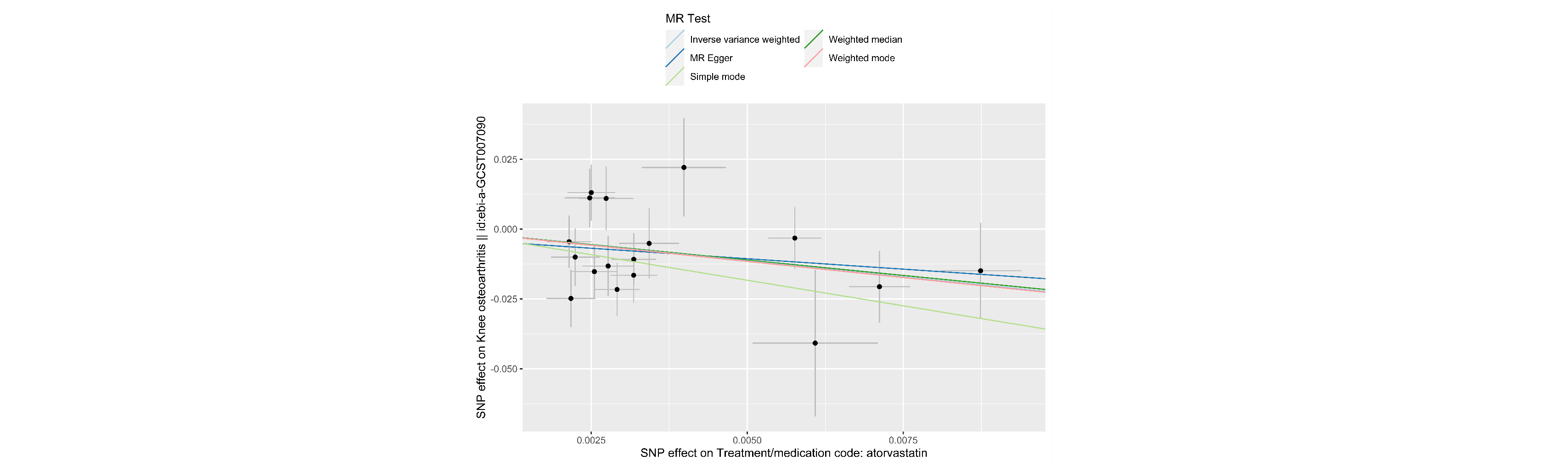


**Figure S6 the scatter plot of the effect of atorvastatin use on knee osteoarthritis**


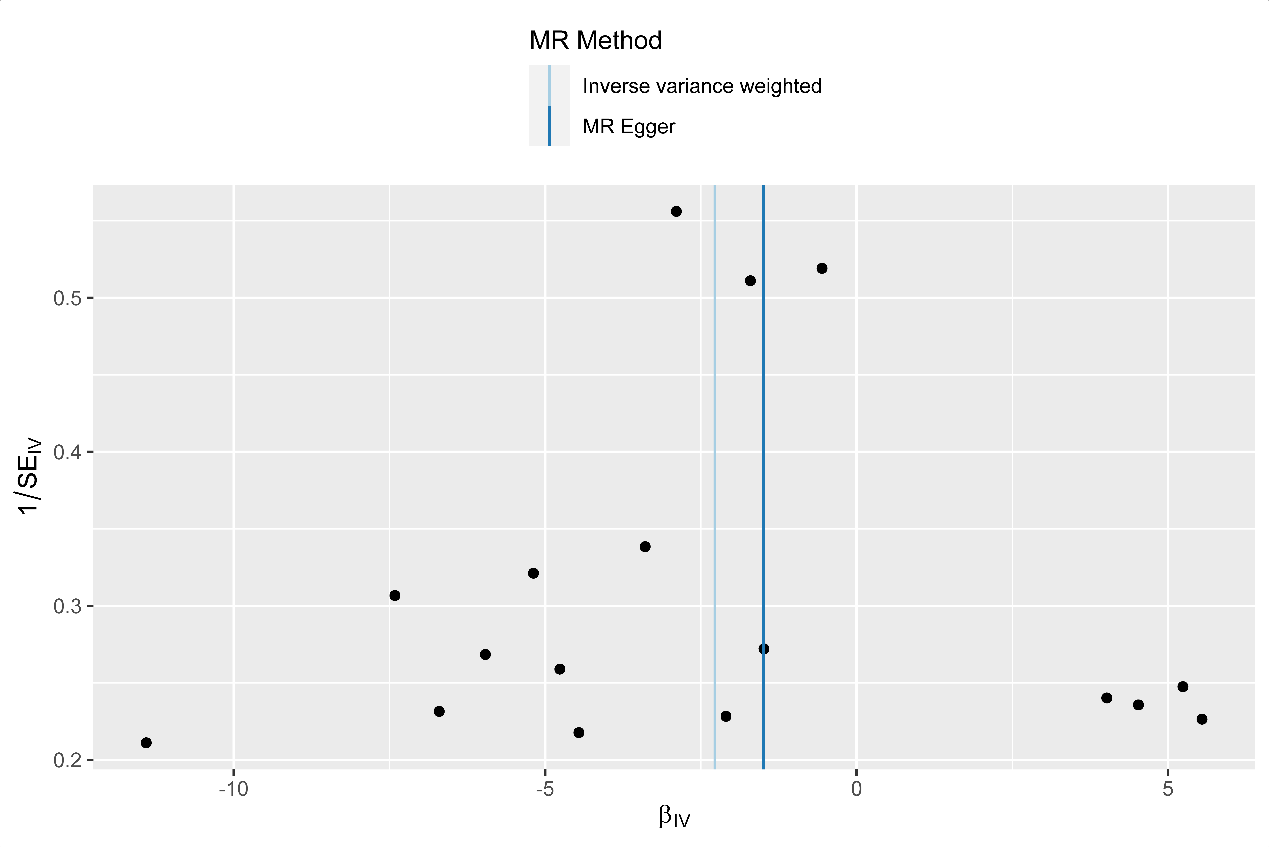


**Figure S7 the funnel plot of the effect of atorvastatin use on knee osteoarthritis**


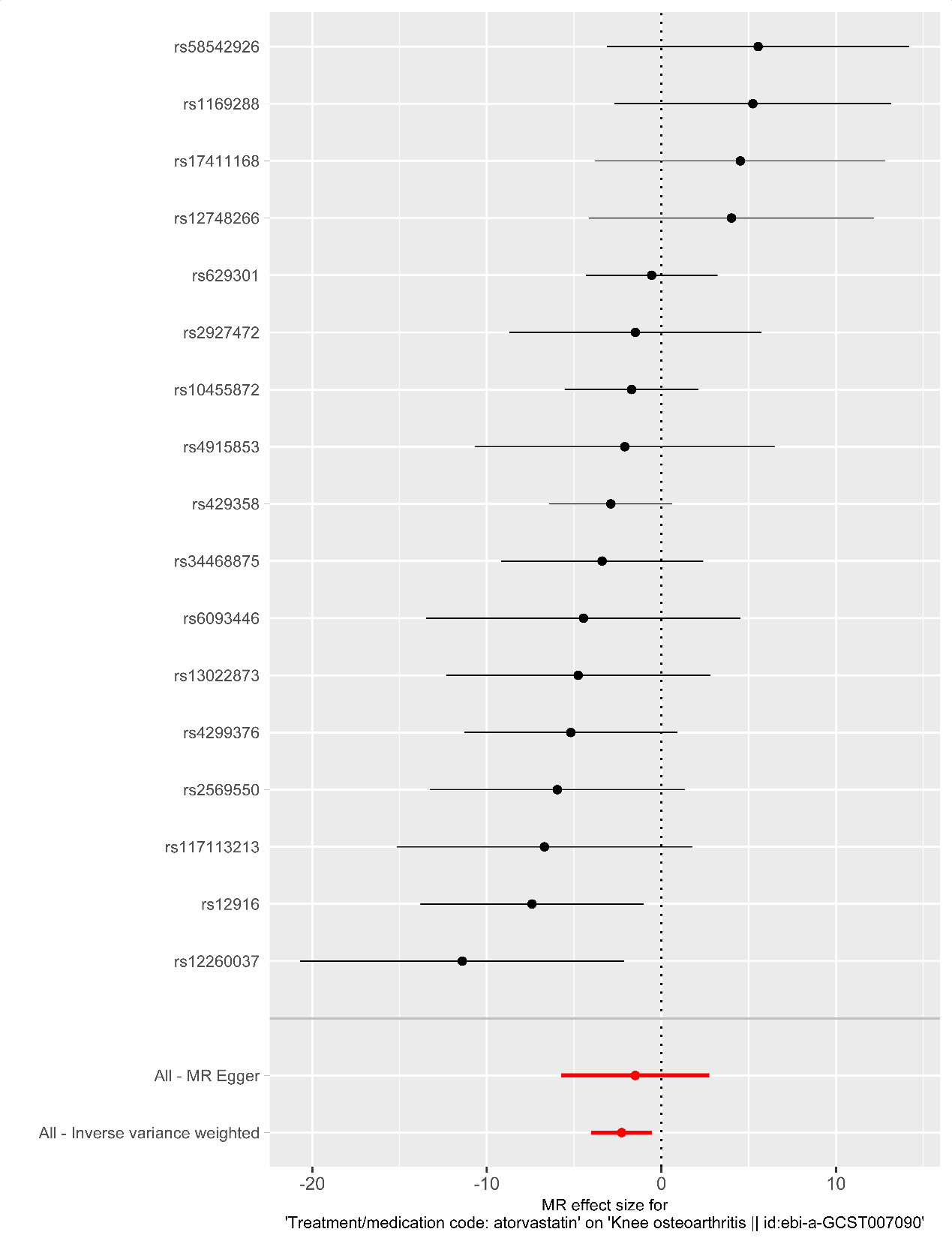


**Figure S8 the forest plot of the effect of atorvastatin use on knee osteoarthritis**


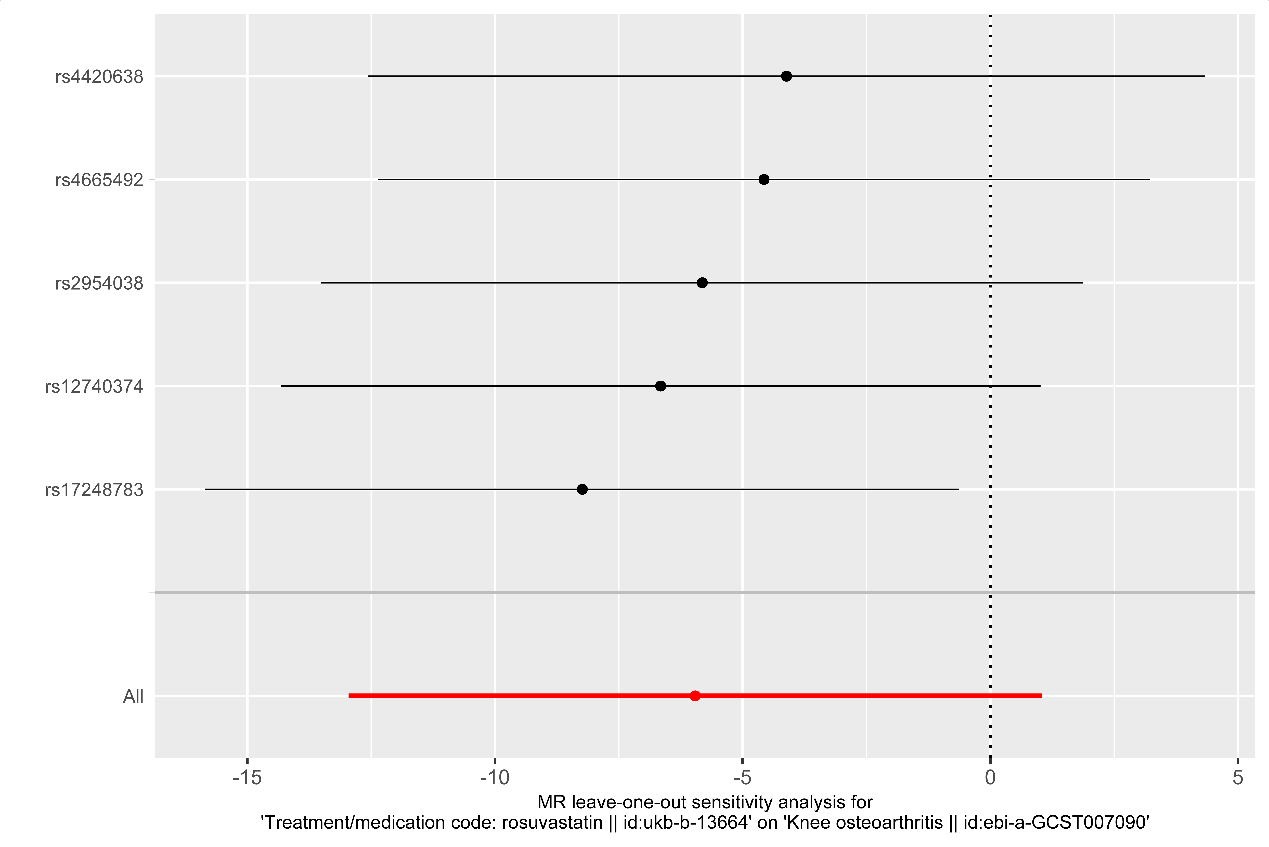


**Figure S9 the leave-one-out plot of the effect of rosuvastatin use on knee osteoarthritis**


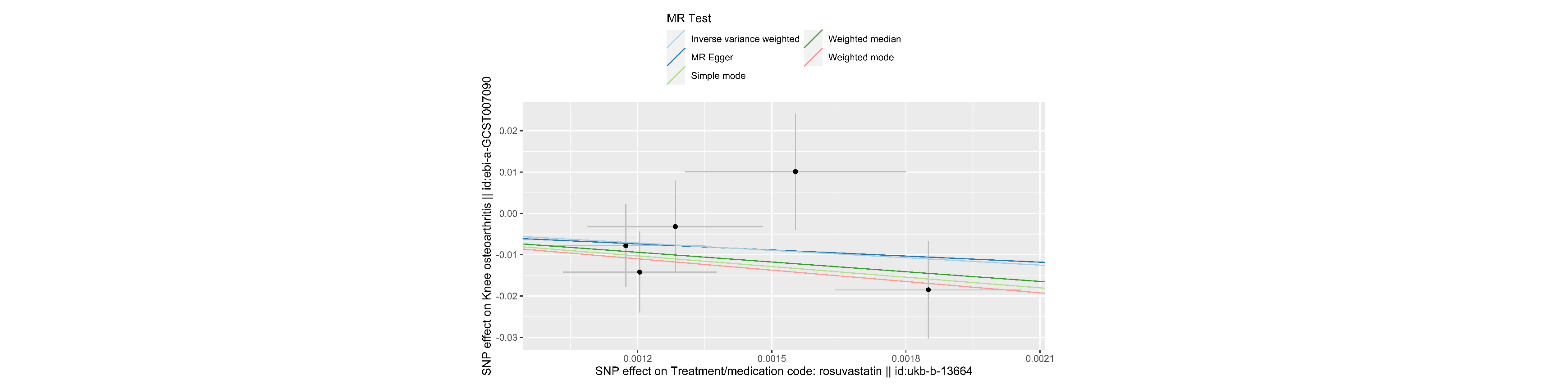


**Figure S10 the scatter plot of the effect of rosuvastatin use on knee osteoarthritis**


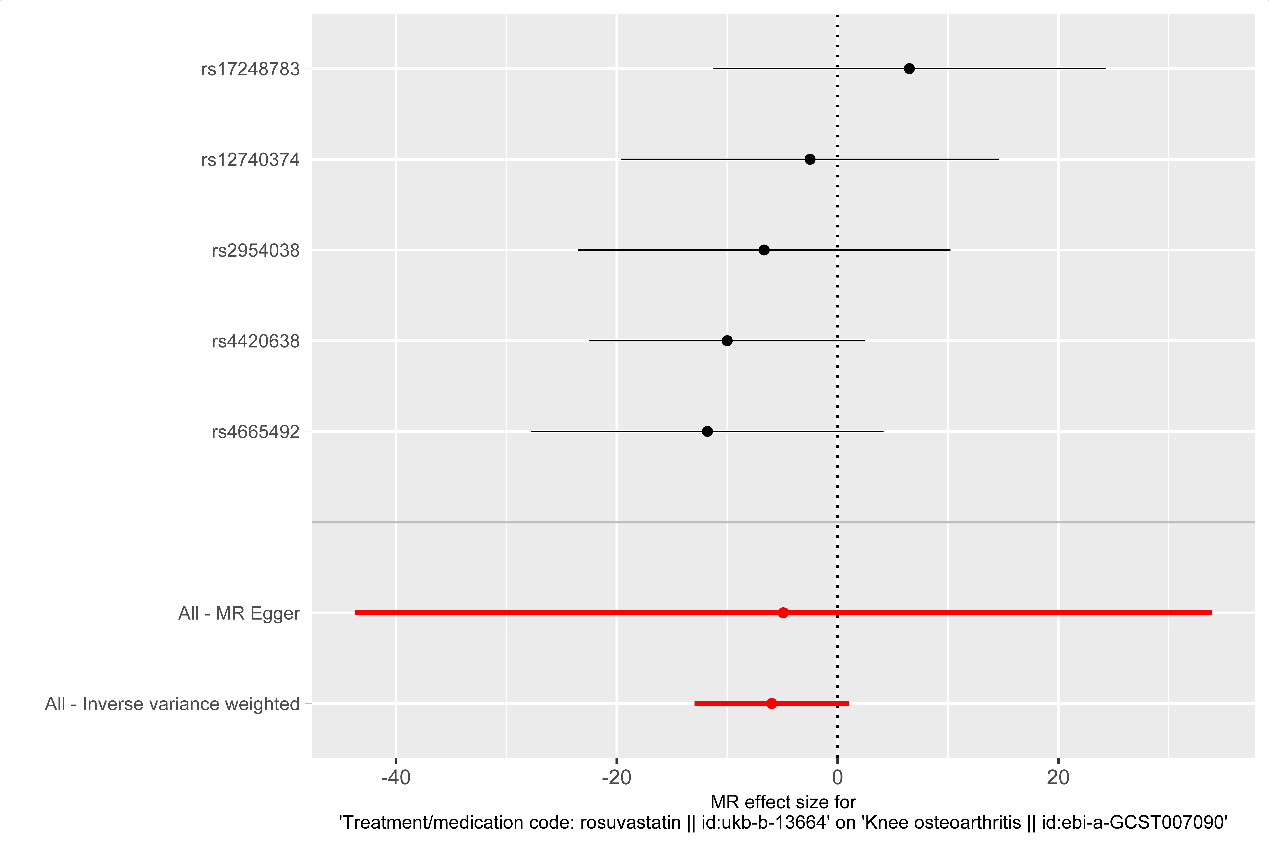


**Figure S11 the forest plot of the effect of rosuvastatin use on knee osteoarthritis**


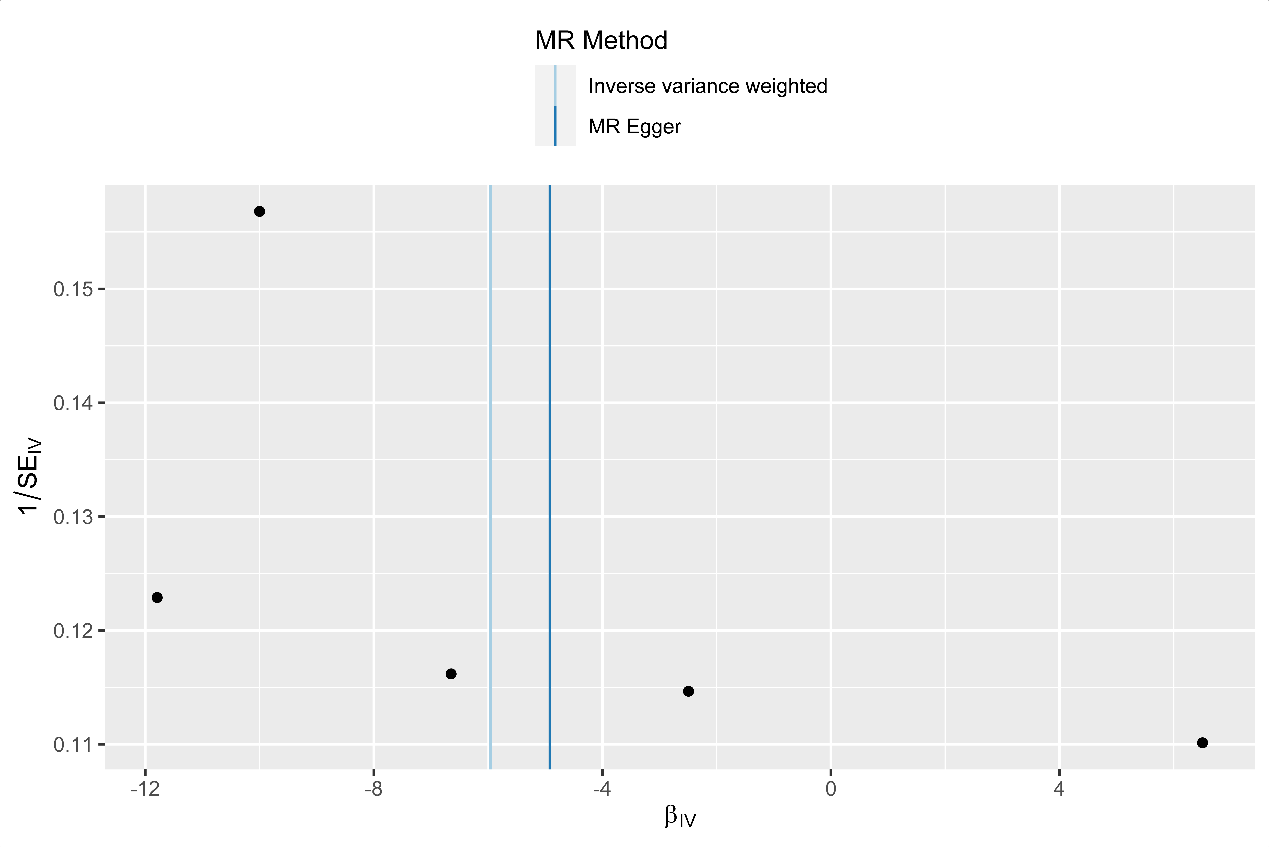


**Figure S12 the funnel plot of the effect of rosuvastatin use on knee osteoarthritis**


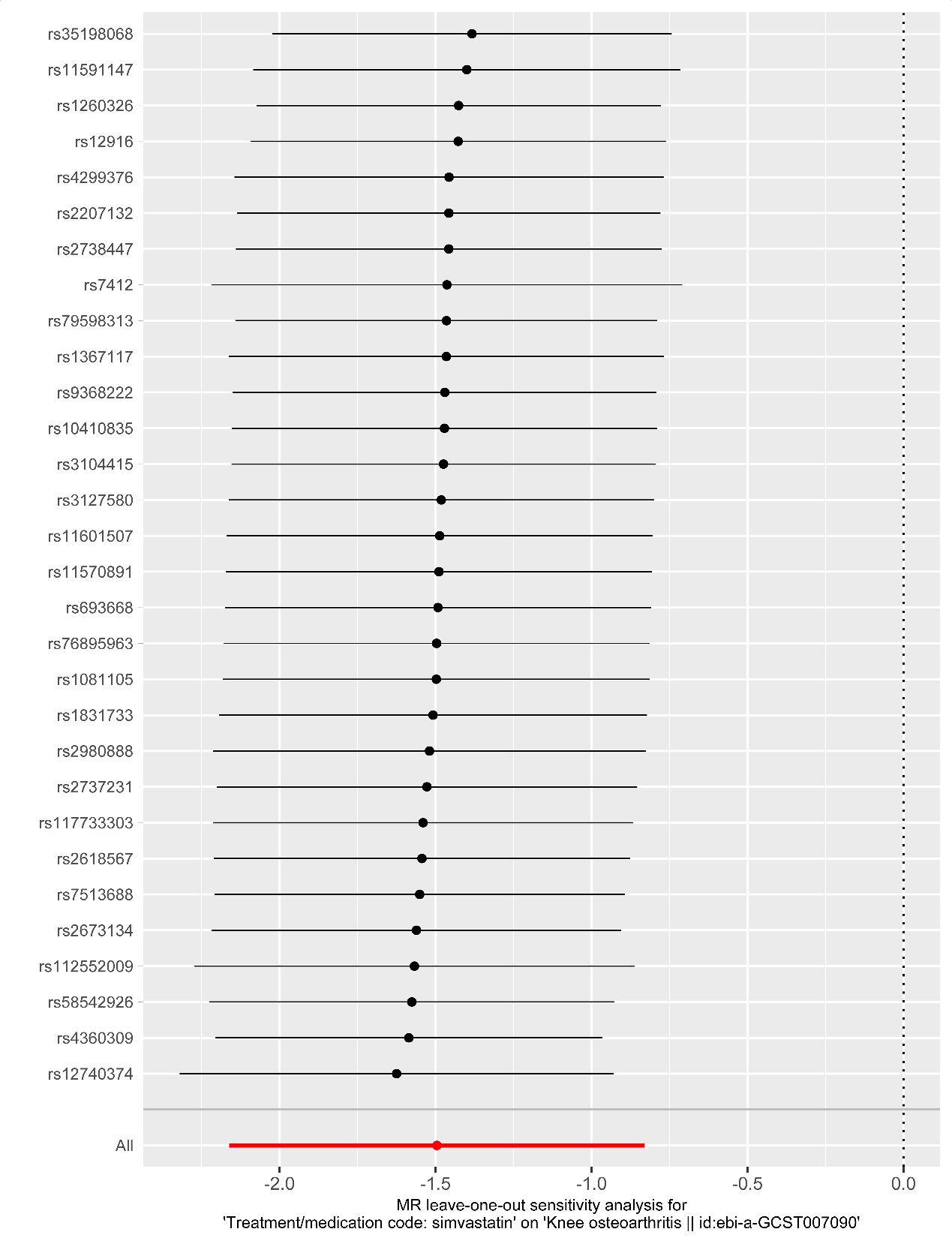


**Figure S13 the leave-one-out plot of the effect of simvastatin use on knee osteoarthritis**


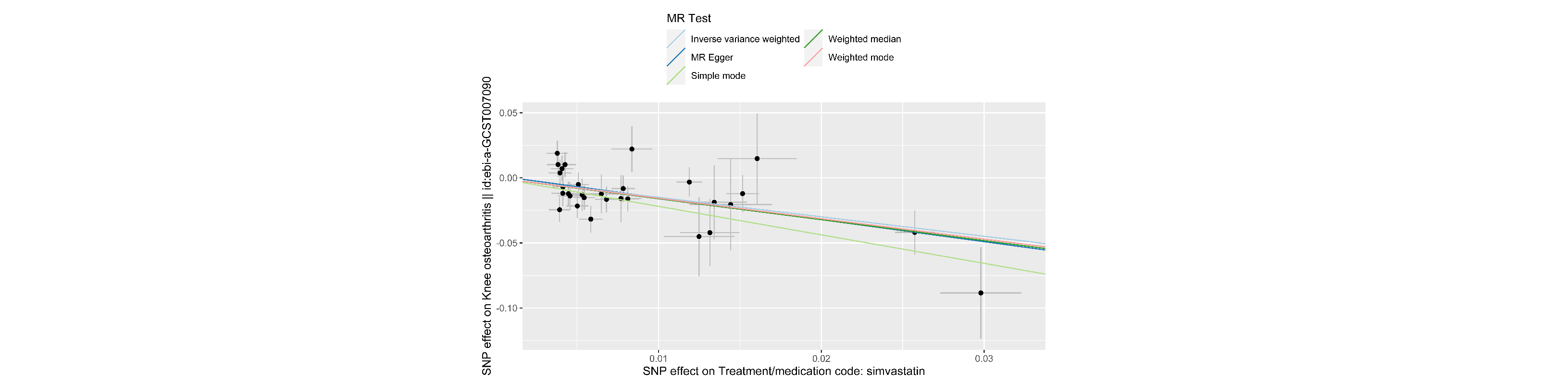


**Figure S14 the scatter plot of the effect of simvastatin use on knee osteoarthritis**


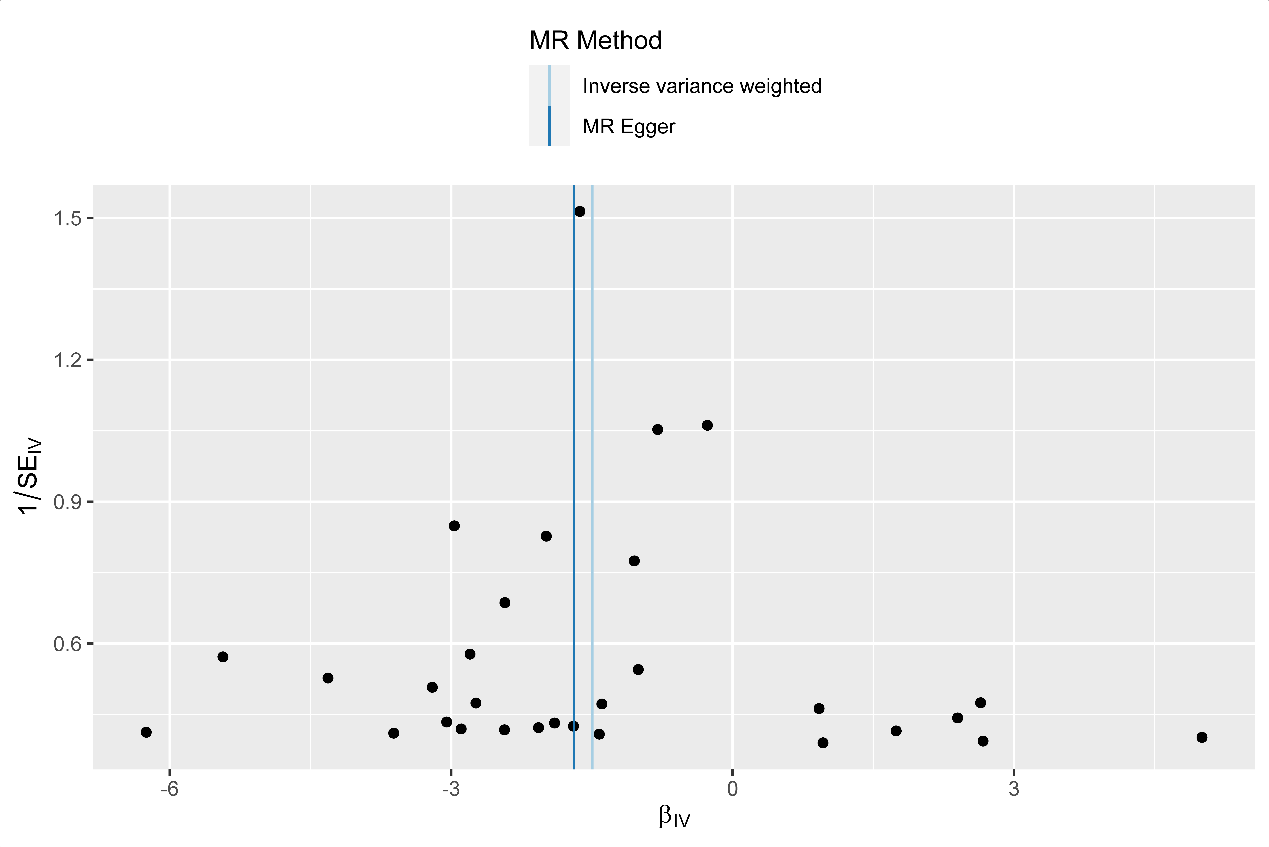


**Figure S15 the funnel plot of the effect of simvastatin use on knee osteoarthritis**


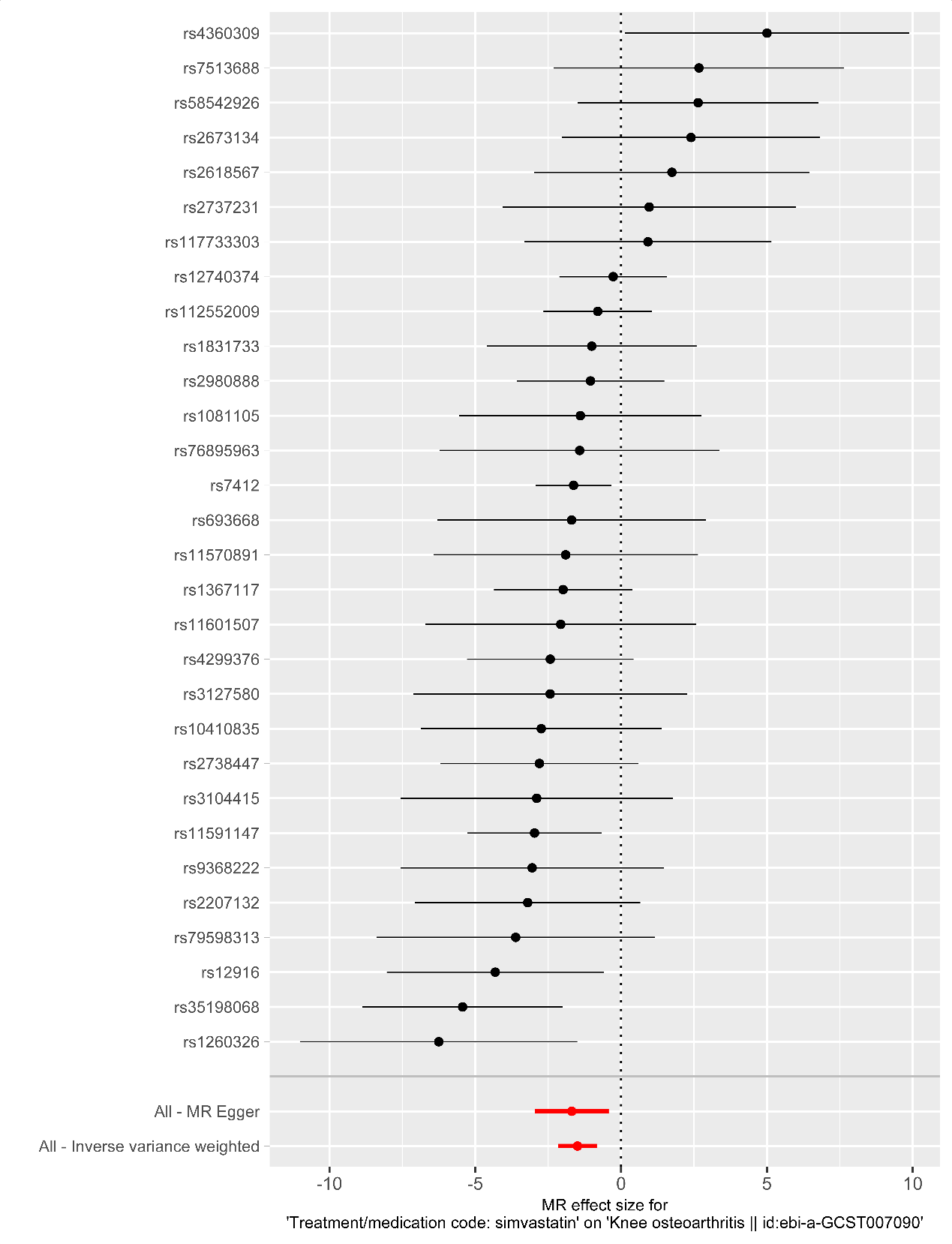


**Figure S16 the forest plot of the effect of simvastatin use on knee osteoarthritis**


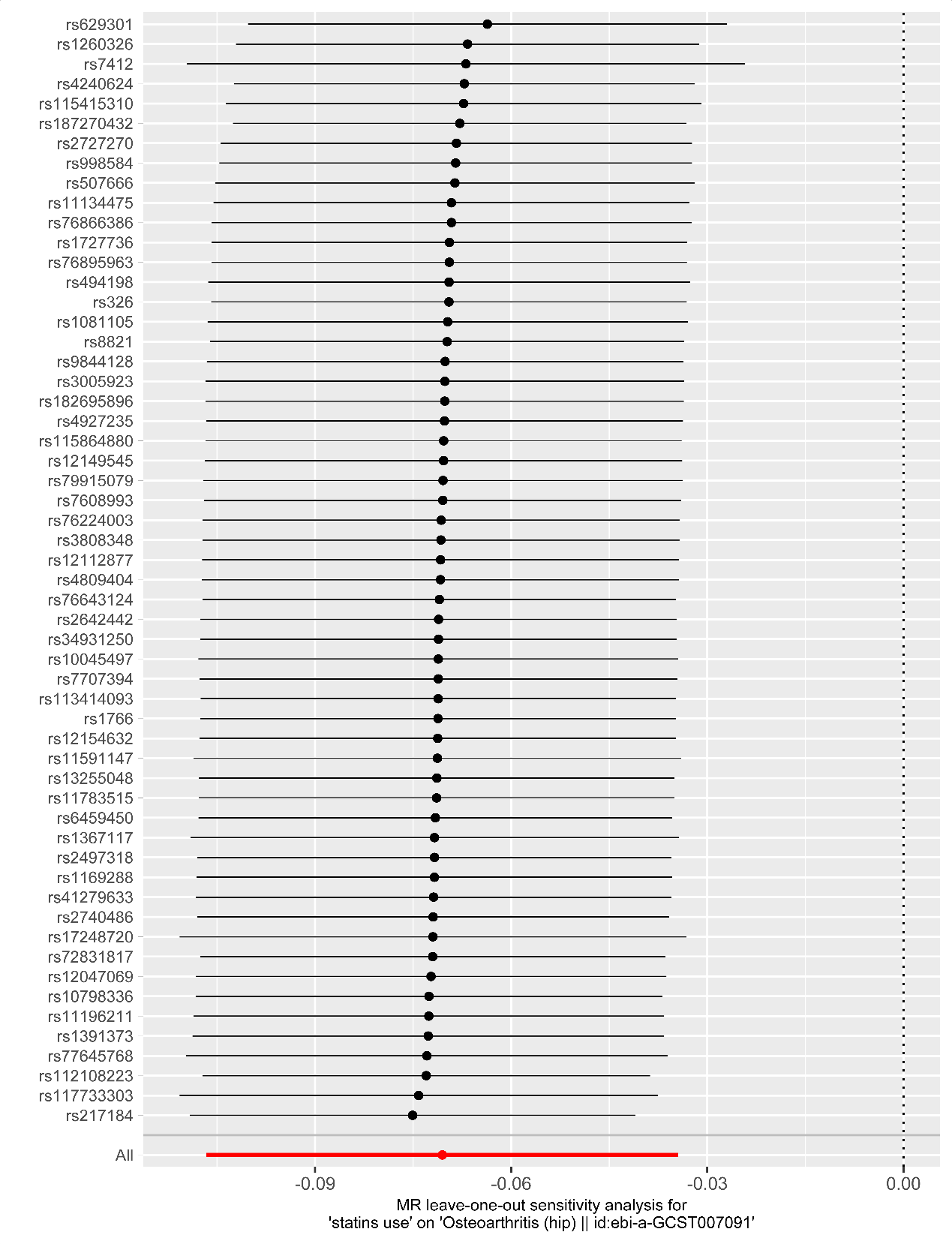


**Figure S17 the leave-one-out plot of the effect of** **statins use on hip osteoarthritis**


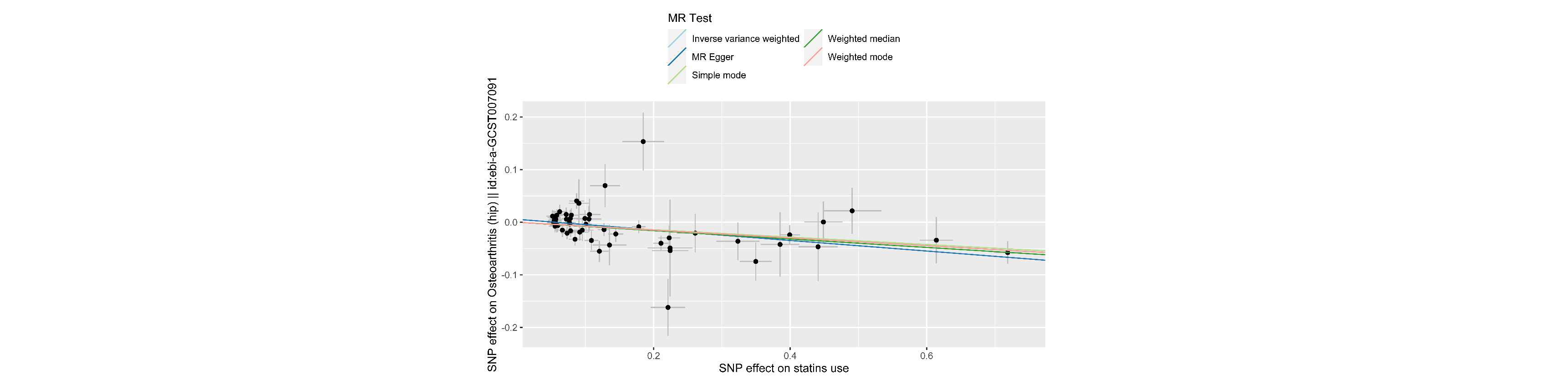


**Figure S18 the scatter plot of the effect of statins use on hip osteoarthritis**


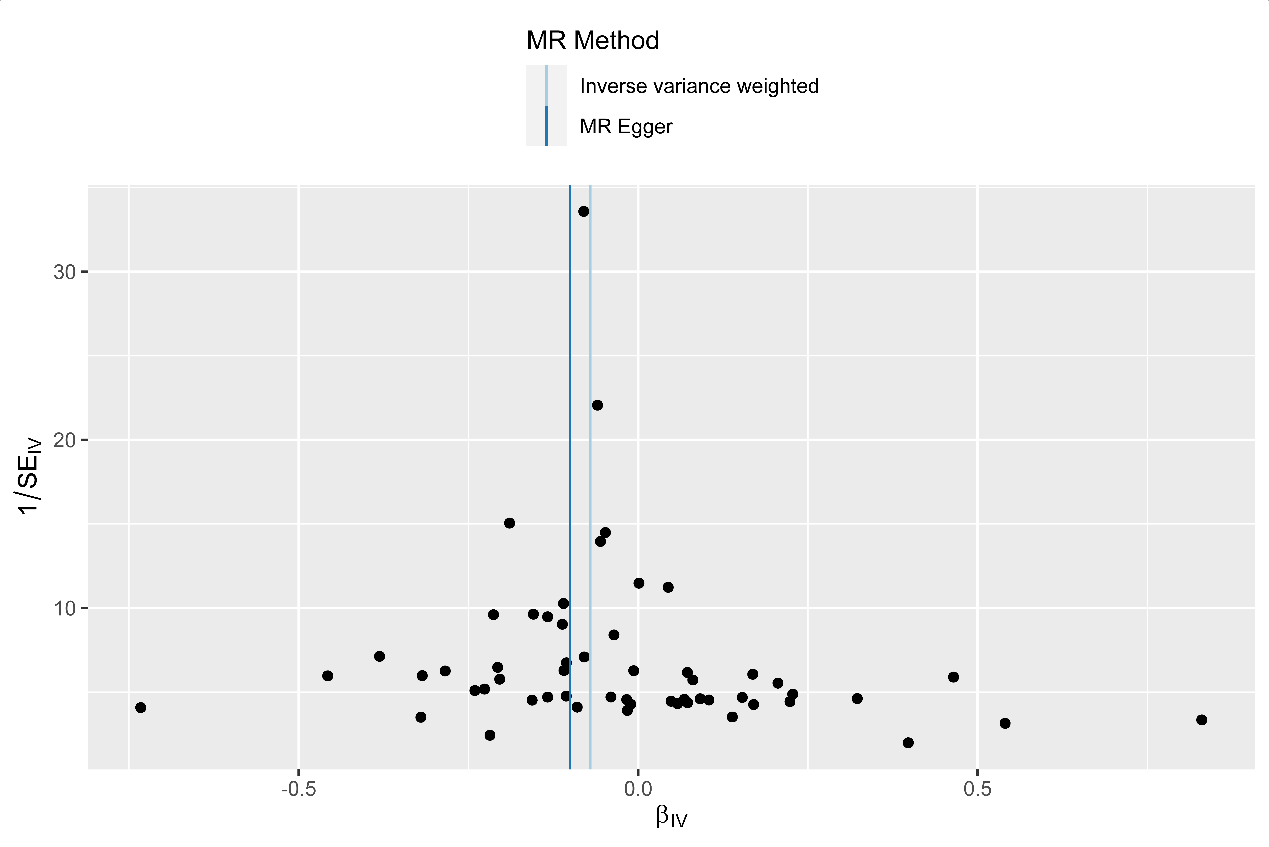


**Figure S19 the funnel plot of the effect of statins use on hip osteoarthritis**


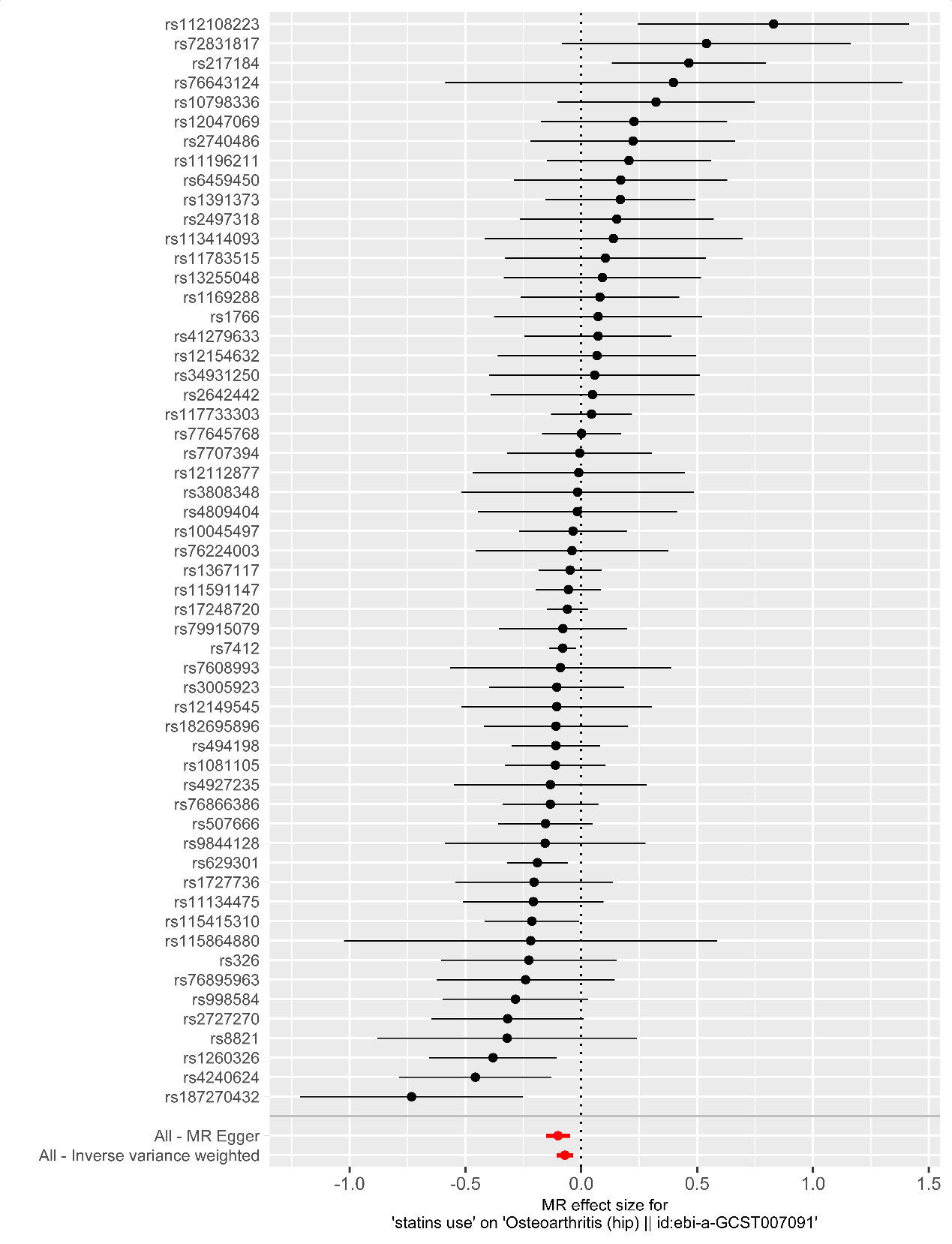


**Figure S20 the forest plot of the effect of statins use on hip osteoarthritis**


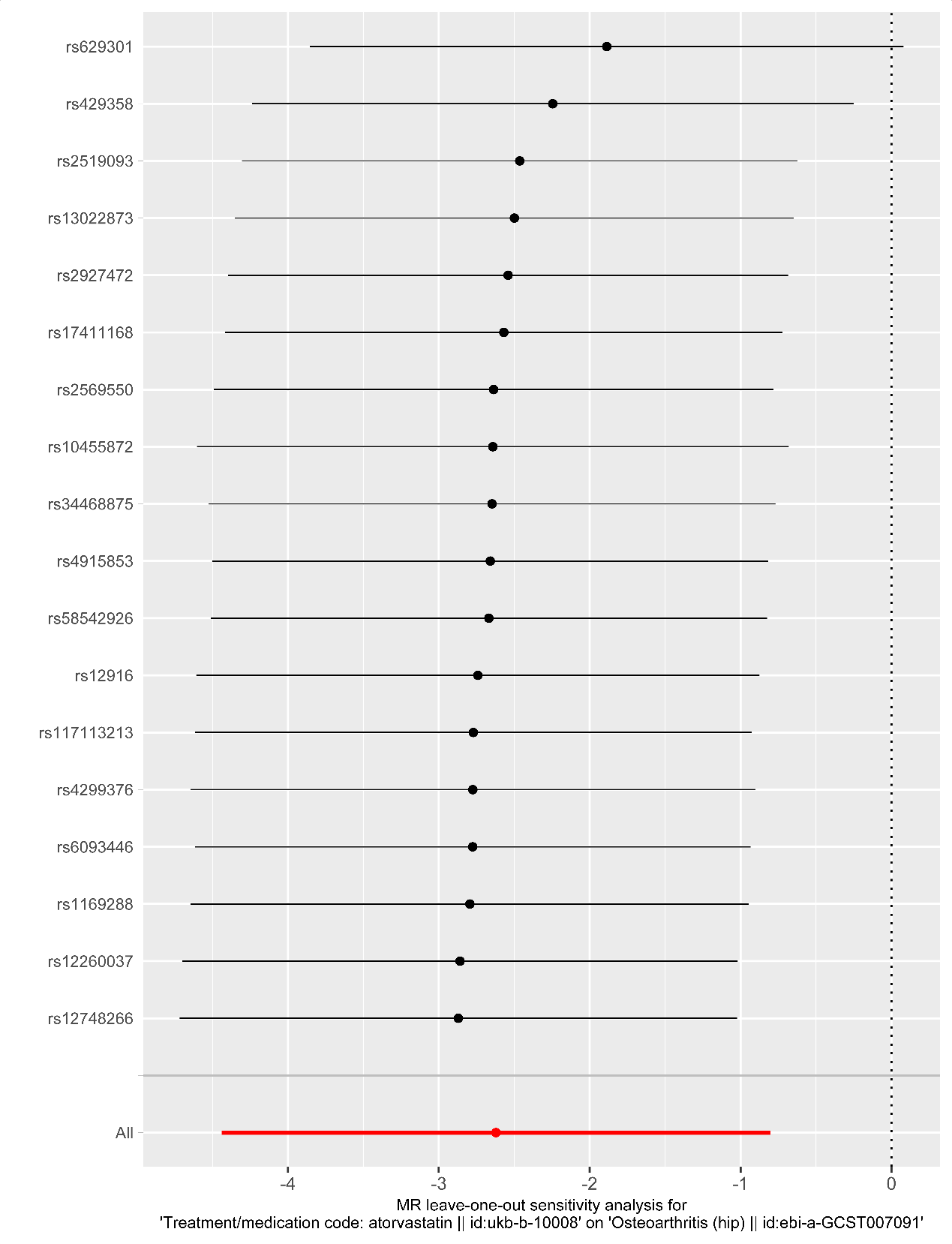


**Figure S21 the leave-one-out plot of the effect of atorvastatin use on hip osteoarthritis**


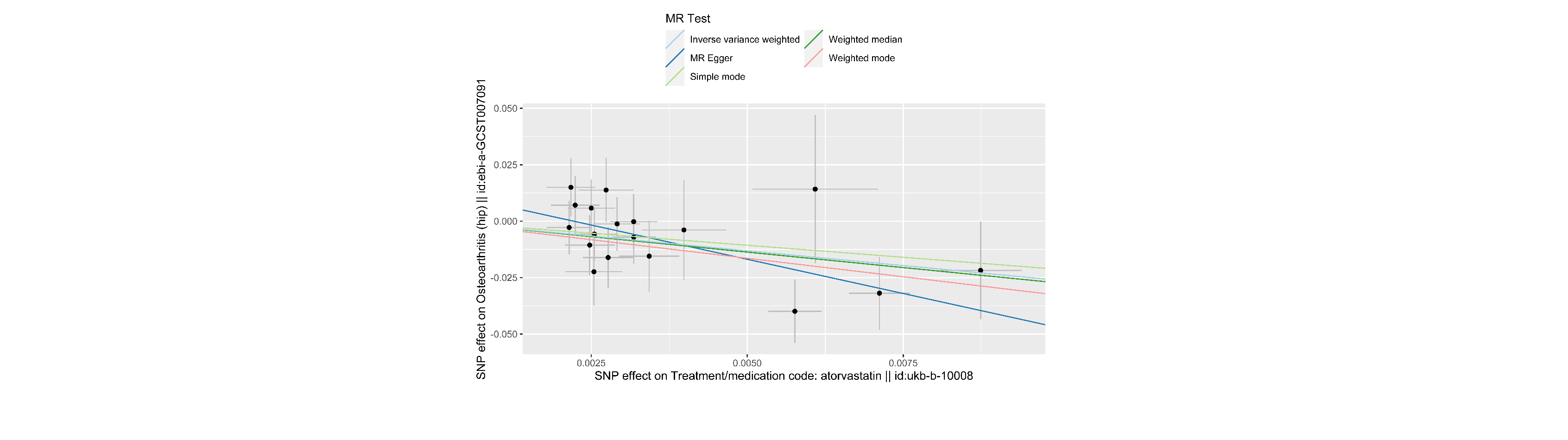


**Figure S22 the scatter plot of the effect of atorvastatin use on hip osteoarthritis**


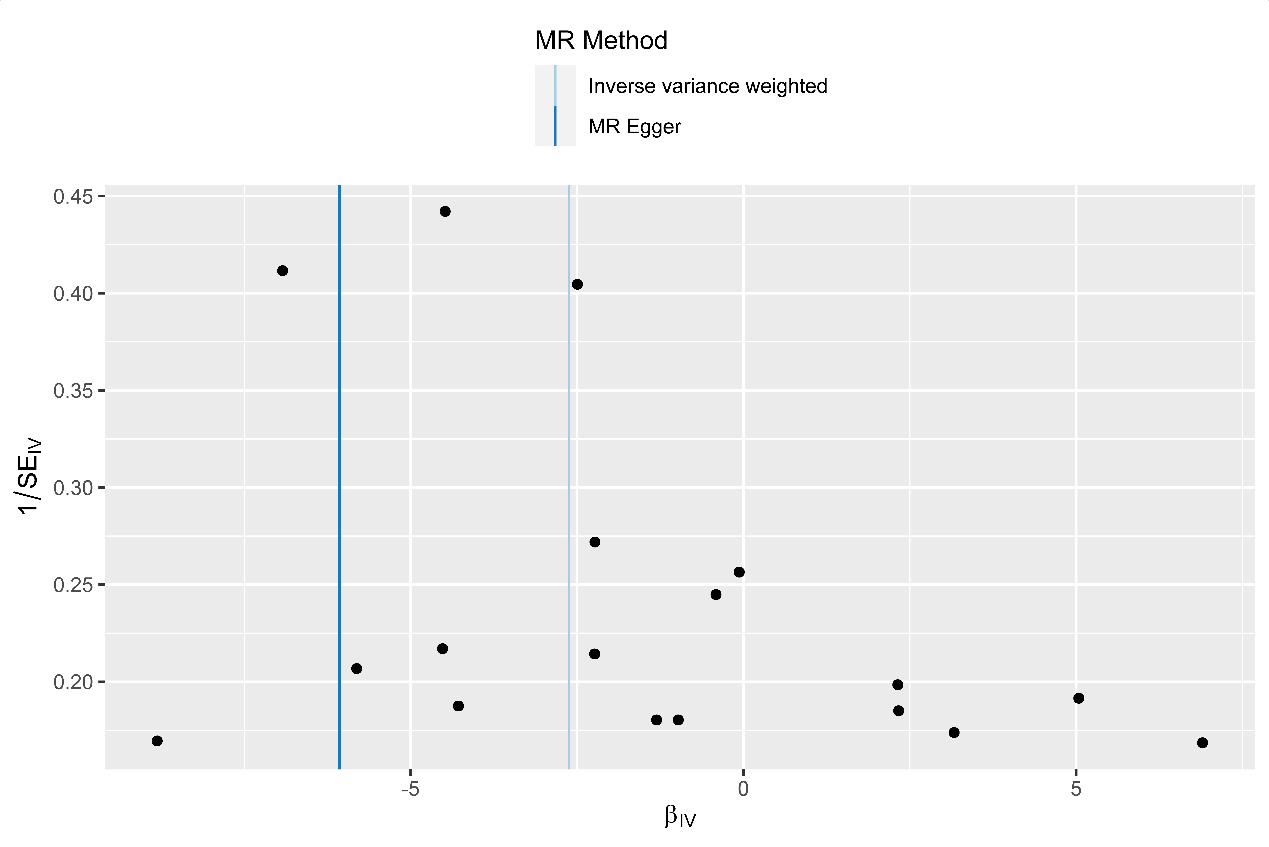


**Figure S23 the funnel plot of the effect of atorvastatin use on hip osteoarthritis**


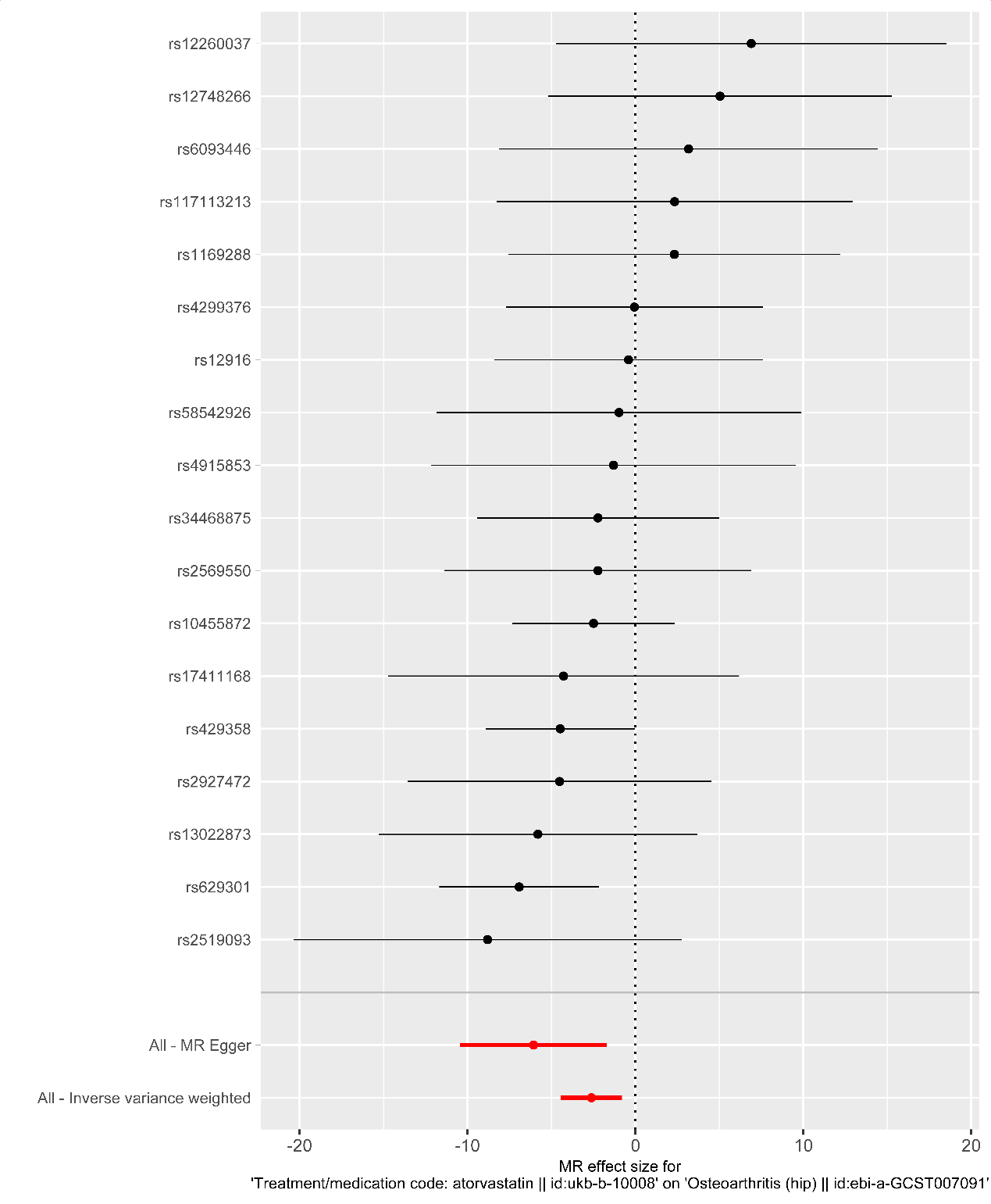


**Figure S24 the forest plot of the effect of atorvastatin use on hip osteoarthritis**


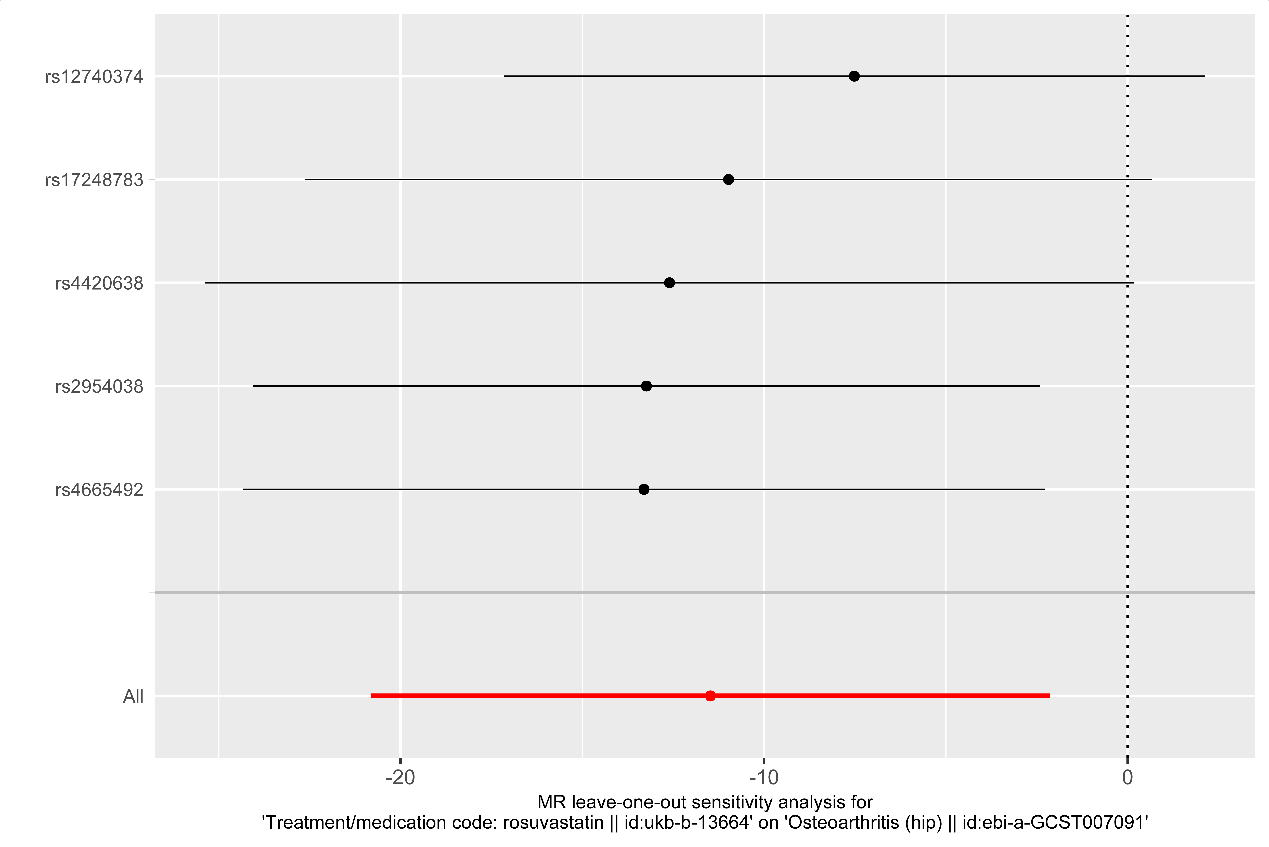


**Figure S25 the leave-one-out plot of the effect of rosuvastatin use on hip osteoarthritis**


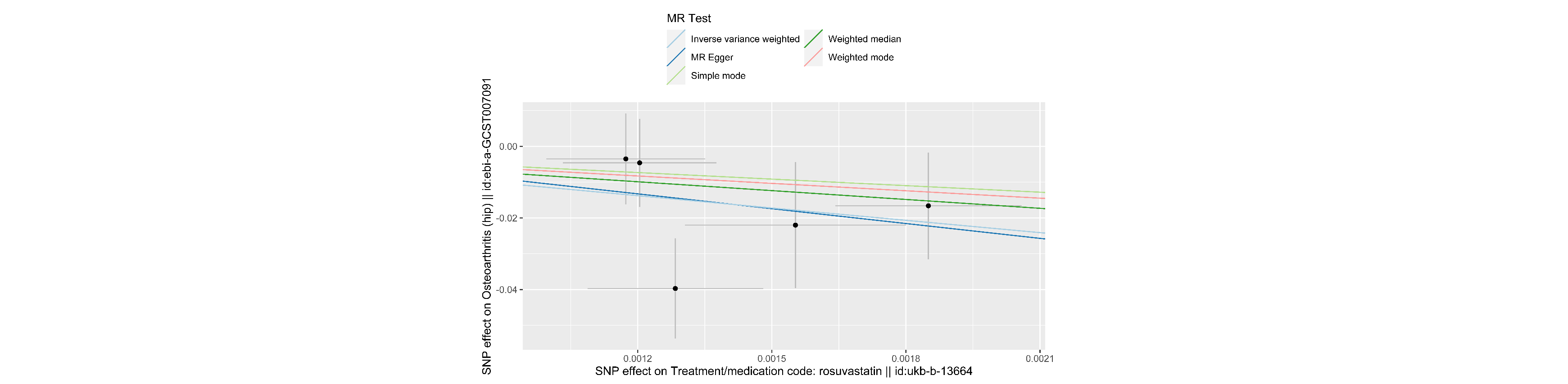


**Figure S26 the scatter plot of the effect of rosuvastatin use on hip osteoarthritis**


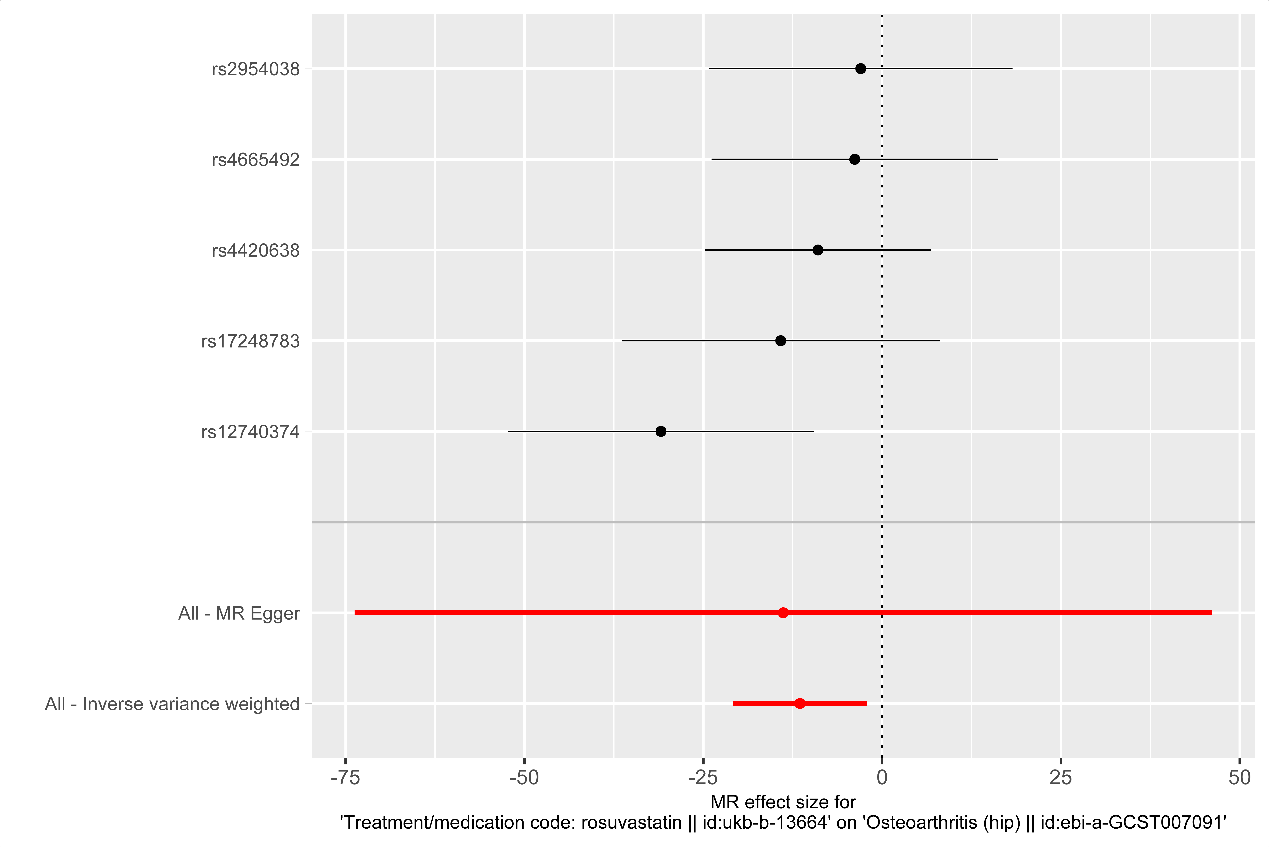


**Figure S27 the forest plot of the effect of rosuvastatin use on hip osteoarthritis**


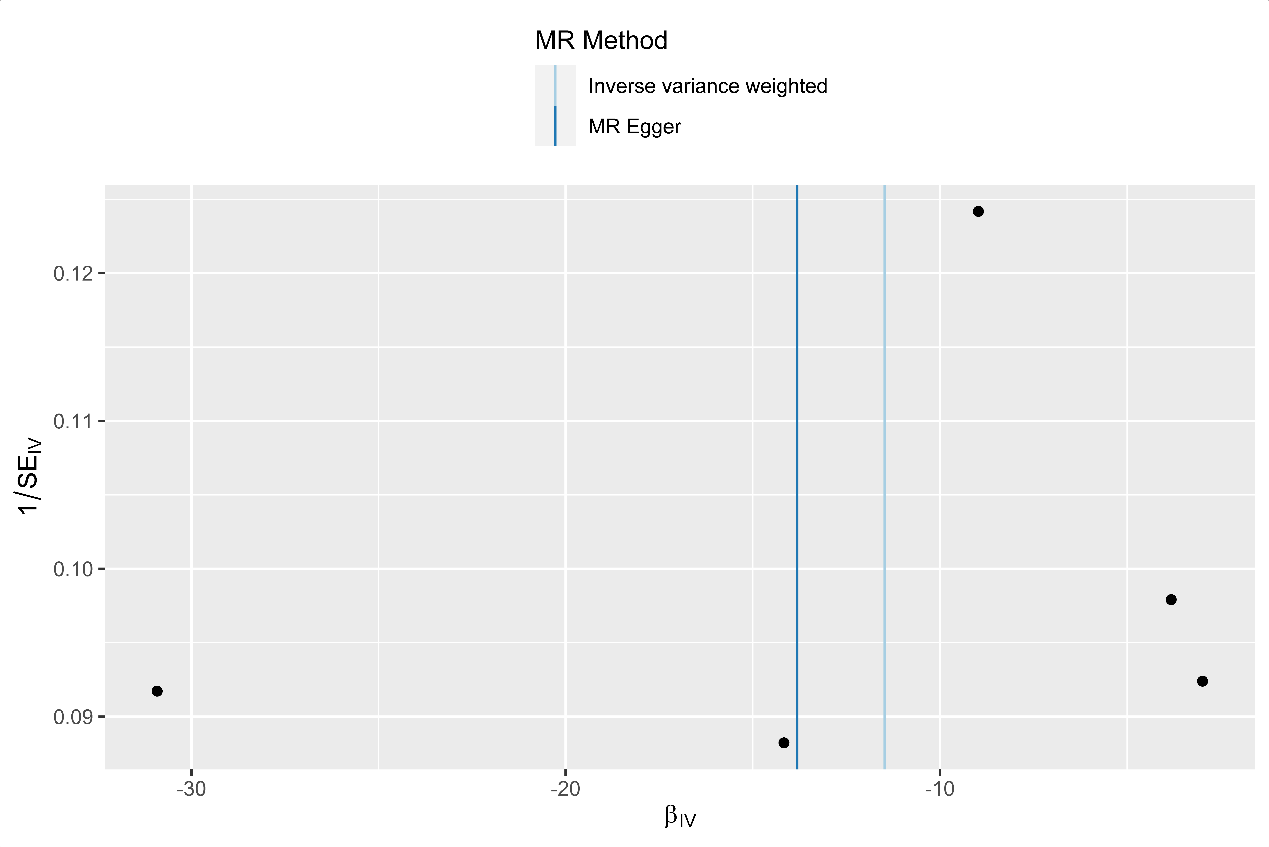


**Figure S28 the funnel plot of the effect of rosuvastatin use on hip osteoarthritis**


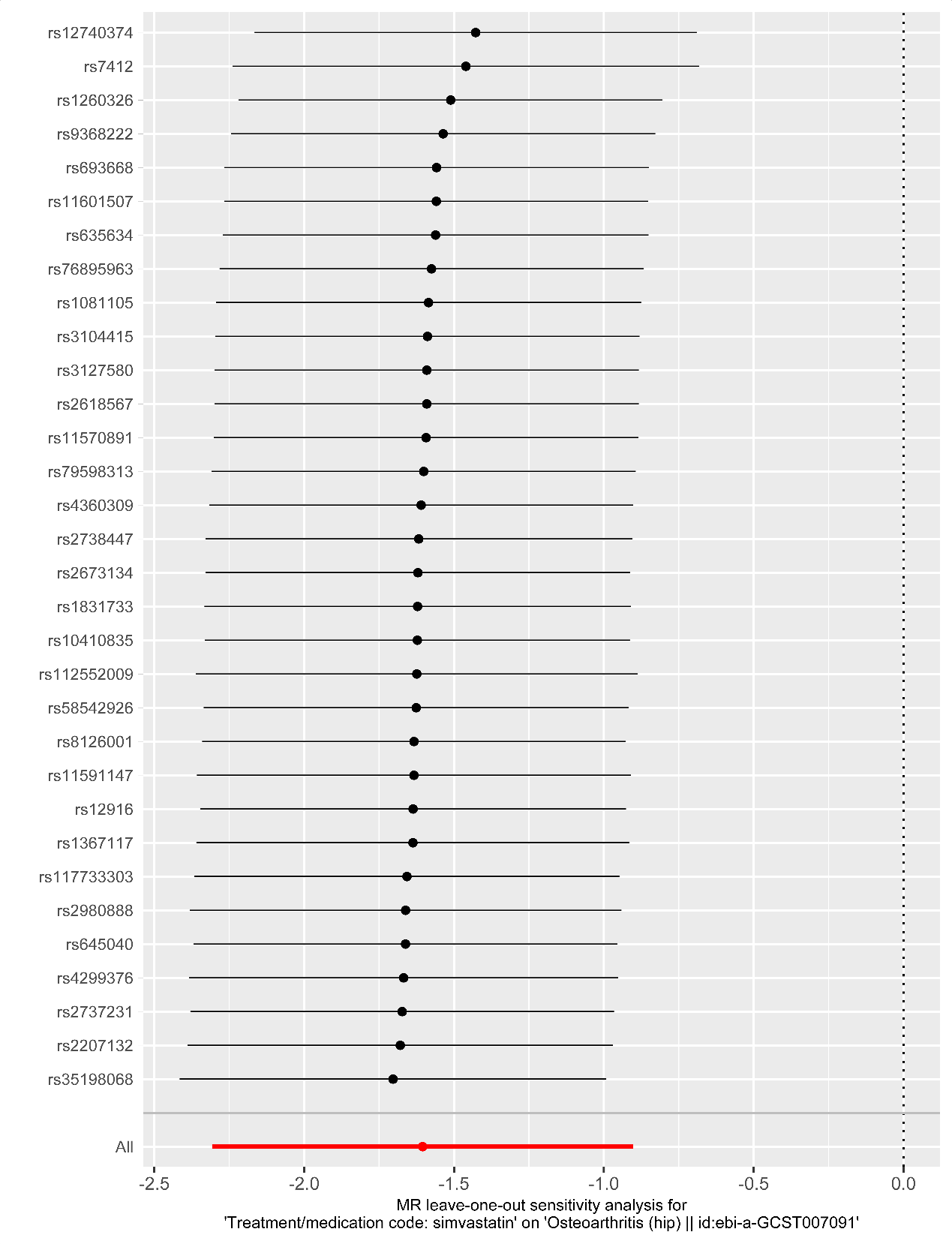


**Figure S29 the leave-one-out plot of the effect of simvastatin use on hip osteoarthritis**


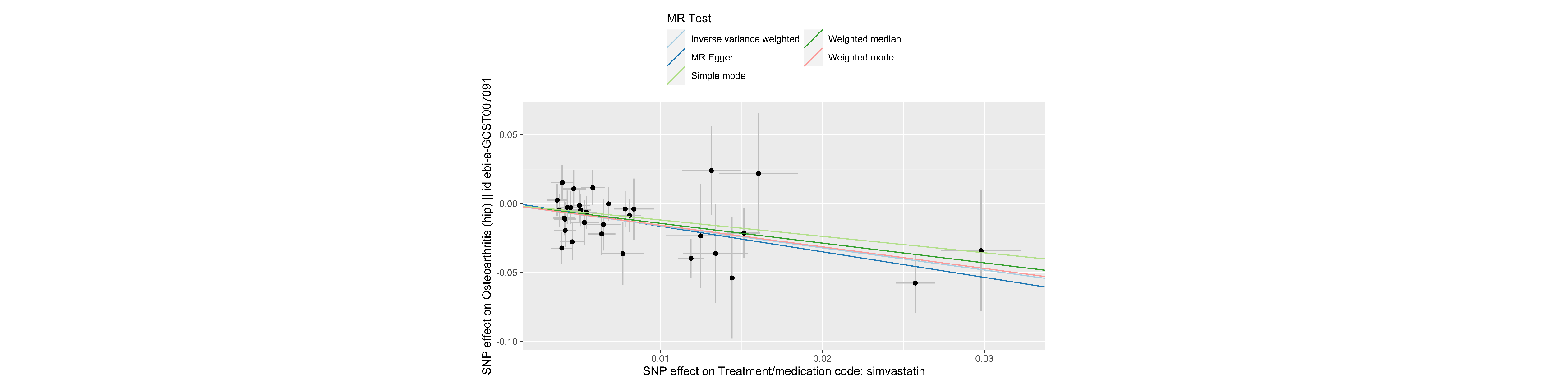


**Figure S30 the scatter plot of the effect of simvastatin use on hip osteoarthritis**


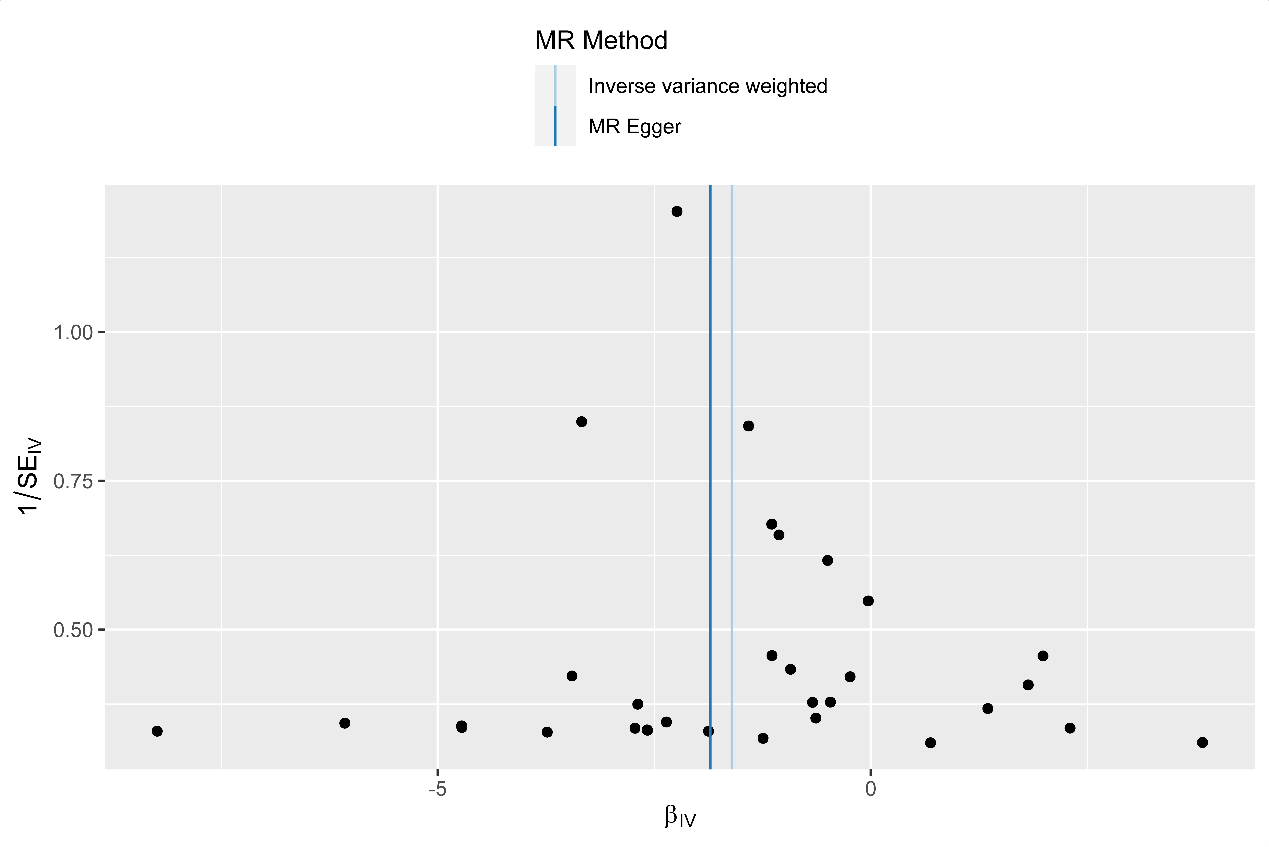


**Figure S31 the funnel plot of the effect of simvastatin use on hip osteoarthritis**


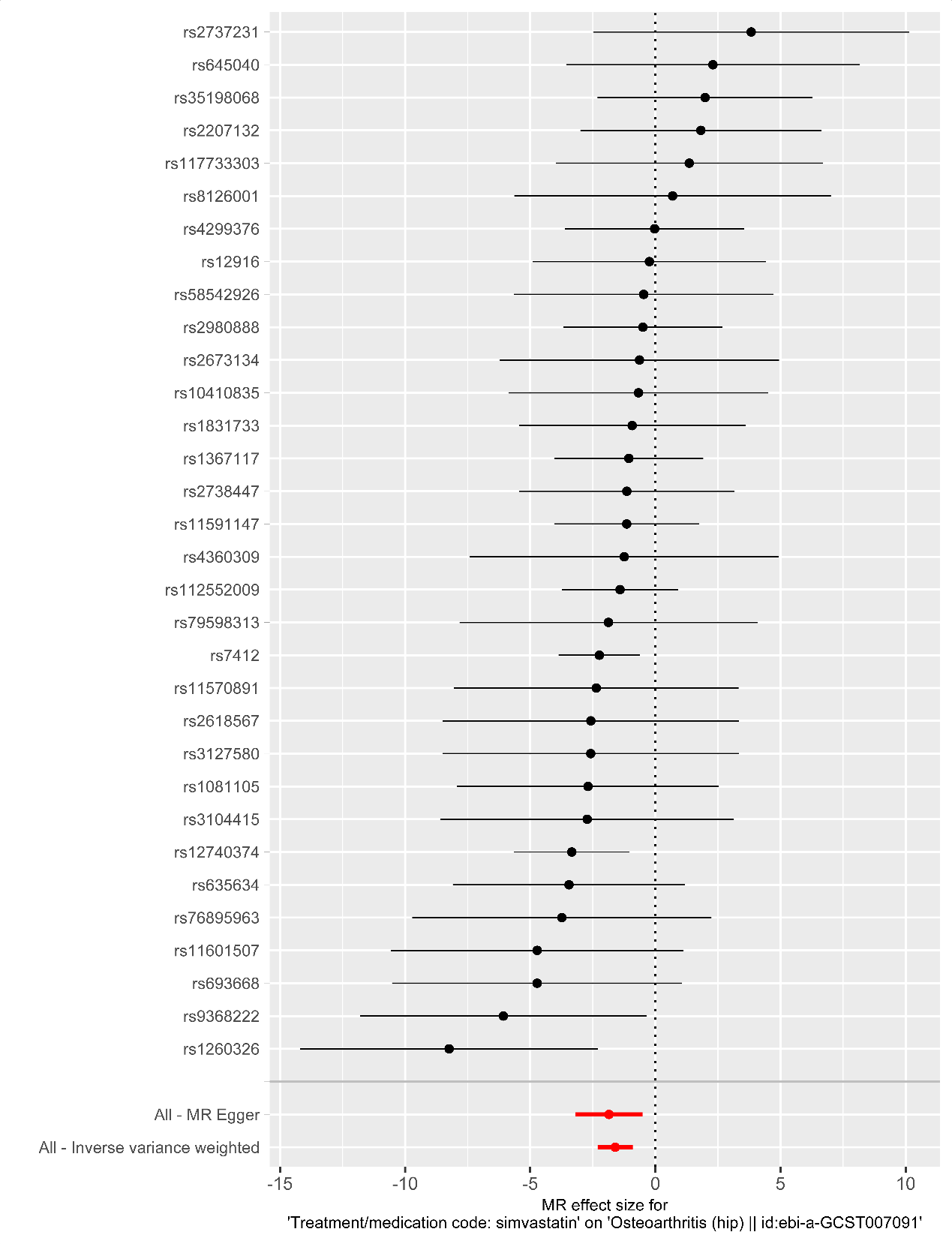


**Figure S32 the forest plot of the effect of simvastatin use on hip osteoarthritis**

**Table S1** Genome-wide significant SNPs associated with statins use in knee osteoarthritis

| SNP | EA | OA | Beta | SE | P | F-statistic |
| --- | --- | --- | --- | --- | --- | --- |
| rs10045497 | A | C | 0.1008 | 0.009 | 4.97E-29 | 125.44 |
| rs10798336 | A | G | 0.0623 | 0.0113 | 3.09E-08 | 30.39619 |
| rs1081105 | C | A | 0.3234 | 0.0315 | 8.81E-25 | 105.4044 |
| rs11134475 | G | A | 0.0783 | 0.0095 | 2.25E-16 | 67.9323 |
| rs11196211 | C | A | 0.0719 | 0.0117 | 7.59E-10 | 37.7647 |
| rs112108223 | A | G | -0.1847 | 0.0306 | 1.59E-09 | 36.43266 |
| rs113414093 | A | G | 0.106 | 0.0162 | 5.64E-11 | 42.8136 |
| rs115415310 | C | T | 0.3496 | 0.0235 | 3.91E-50 | 221.3131 |
| rs115864880 | A | G | -0.224 | 0.0328 | 7.98E-12 | 46.63891 |
| rs11591147 | T | G | -0.6142 | 0.0245 | 6.62E-139 | 628.4742 |
| rs1169288 | C | A | 0.0721 | 0.0093 | 8.54E-15 | 60.10417 |
| rs117733303 | G | A | 0.4908 | 0.0427 | 1.56E-30 | 132.1154 |
| rs11783515 | G | A | -0.0566 | 0.0092 | 8.88E-10 | 37.84924 |
| rs12047069 | C | A | -0.058 | 0.0091 | 2.03E-10 | 40.62311 |
| rs12112877 | C | T | 0.0547 | 0.0097 | 1.73E-08 | 31.8003 |
| rs12149545 | A | G | -0.0595 | 0.01 | 2.82E-09 | 35.4025 |
| rs12154632 | T | C | -0.0544 | 0.0093 | 5.60E-09 | 34.21621 |
| rs1260326 | C | T | -0.0848 | 0.0094 | 1.58E-19 | 81.38343 |
| rs13255048 | A | G | 0.0778 | 0.0116 | 2.14E-11 | 44.98246 |
| rs1367117 | A | G | 0.1782 | 0.0099 | 3.52E-72 | 324 |
| rs1391373 | C | T | 0.0794 | 0.0108 | 2.24E-13 | 54.04973 |
| rs17248720 | T | C | -0.3993 | 0.0149 | 2.29E-157 | 718.1681 |
| rs1727736 | C | T | 0.0917 | 0.0128 | 6.78E-13 | 51.32379 |
| rs1766 | G | A | 0.055 | 0.0097 | 1.64E-08 | 32.15007 |
| rs182695896 | C | A | 0.3852 | 0.0283 | 3.40E-42 | 185.2677 |
| rs187270432 | A | G | 0.221 | 0.0252 | 1.59E-18 | 76.91012 |
| rs217184 | C | T | -0.0872 | 0.0109 | 1.61E-15 | 64 |
| rs2497318 | T | C | 0.0548 | 0.009 | 1.14E-09 | 37.07457 |
| rs2642442 | T | C | 0.0557 | 0.0099 | 1.64E-08 | 31.65483 |
| rs2727270 | T | C | -0.1088 | 0.0101 | 5.07E-27 | 116.042 |
| rs2740486 | G | T | -0.0519 | 0.009 | 9.28E-09 | 33.25444 |
| rs3005923 | A | G | -0.4409 | 0.0288 | 8.86E-53 | 234.3663 |
| rs326 | G | A | -0.0663 | 0.0102 | 8.24E-11 | 42.25 |
| rs34931250 | T | C | 0.1052 | 0.0181 | 6.32E-09 | 33.78114 |
| rs3808348 | T | C | -0.0563 | 0.01 | 1.72E-08 | 31.6969 |
| rs41279633 | T | G | 0.0993 | 0.011 | 1.74E-19 | 81.49165 |
| rs4240624 | A | G | 0.1205 | 0.0135 | 3.94E-19 | 79.67215 |
| rs4809404 | G | A | -0.0533 | 0.0091 | 4.10E-09 | 34.30612 |
| rs4927235 | G | A | 0.0555 | 0.0091 | 1.00E-09 | 37.19659 |
| rs494198 | A | C | 0.1273 | 0.009 | 5.58E-45 | 200.0653 |
| rs59888912 | G | A | -0.0561 | 0.0102 | 3.82E-08 | 30.25 |
| rs629301 | T | G | 0.2107 | 0.0109 | 1.27E-82 | 373.6595 |
| rs6459450 | C | T | -0.0541 | 0.0092 | 4.85E-09 | 34.57951 |
| rs72831817 | T | C | -0.1288 | 0.0221 | 5.82E-09 | 33.96622 |
| rs7412 | T | C | -0.7187 | 0.0203 | 1.00E-200 | 1253.439 |
| rs7608993 | C | T | -0.0579 | 0.01 | 8.06E-09 | 33.5241 |
| rs76224003 | G | A | 0.0772 | 0.0122 | 2.66E-10 | 40.04192 |
| rs76643124 | A | G | -0.0905 | 0.0149 | 1.17E-09 | 36.89136 |
| rs76866386 | C | T | -0.2227 | 0.0162 | 5.02E-43 | 188.9776 |
| rs76895963 | G | T | -0.2241 | 0.0269 | 8.15E-17 | 69.40315 |
| rs7707394 | A | G | 0.0765 | 0.0093 | 1.29E-16 | 67.66389 |
| rs77645768 | A | G | 0.4487 | 0.028 | 6.52E-58 | 256.8006 |
| rs79915079 | A | G | -0.2609 | 0.0265 | 7.55E-23 | 96.9296 |
| rs8821 | T | C | -0.1353 | 0.0247 | 4.41E-08 | 30.00556 |
| rs9844128 | T | C | -0.0954 | 0.0164 | 6.16E-09 | 33.83834 |

Abbreviation: SNP, single nucleotide polymorphism; EA, effect allele; OA, other allele; Beta, genetic effects of SNP on ulcerative colitis; SE, standard error

**Table S2** Genome-wide significant SNPs associated with statins use in hip osteoarthritis

| SNP | EA | OA | Beta | SE | P | F-statistic |
| --- | --- | --- | --- | --- | --- | --- |
| rs10045497 | A | C | 0.1008 | 0.009 | 4.97E-29 | 125.44 |
| rs10798336 | A | G | 0.0623 | 0.0113 | 3.09E-08 | 30.39619 |
| rs1081105 | C | A | 0.3234 | 0.0315 | 8.81E-25 | 105.4044 |
| rs11134475 | G | A | 0.0783 | 0.0095 | 2.25E-16 | 67.9323 |
| rs11196211 | C | A | 0.0719 | 0.0117 | 7.59E-10 | 37.7647 |
| rs112108223 | A | G | -0.1847 | 0.0306 | 1.59E-09 | 36.43266 |
| rs113414093 | A | G | 0.106 | 0.0162 | 5.64E-11 | 42.8136 |
| rs115415310 | C | T | 0.3496 | 0.0235 | 3.91E-50 | 221.3131 |
| rs115864880 | A | G | -0.224 | 0.0328 | 7.98E-12 | 46.63891 |
| rs11591147 | T | G | -0.6142 | 0.0245 | 6.62E-139 | 628.4742 |
| rs1169288 | C | A | 0.0721 | 0.0093 | 8.54E-15 | 60.10417 |
| rs117733303 | G | A | 0.4908 | 0.0427 | 1.56E-30 | 132.1154 |
| rs11783515 | G | A | -0.0566 | 0.0092 | 8.88E-10 | 37.84924 |
| rs12047069 | C | A | -0.058 | 0.0091 | 2.03E-10 | 40.62311 |
| rs12112877 | C | T | 0.0547 | 0.0097 | 1.73E-08 | 31.8003 |
| rs12149545 | A | G | -0.0595 | 0.01 | 2.82E-09 | 35.4025 |
| rs12154632 | T | C | -0.0544 | 0.0093 | 5.60E-09 | 34.21621 |
| rs1260326 | C | T | -0.0848 | 0.0094 | 1.58E-19 | 81.38343 |
| rs13255048 | A | G | 0.0778 | 0.0116 | 2.14E-11 | 44.98246 |
| rs1367117 | A | G | 0.1782 | 0.0099 | 3.52E-72 | 324 |
| rs1391373 | C | T | 0.0794 | 0.0108 | 2.24E-13 | 54.04973 |
| rs17248720 | T | C | -0.3993 | 0.0149 | 2.29E-157 | 718.1681 |
| rs1727736 | C | T | 0.0917 | 0.0128 | 6.78E-13 | 51.32379 |
| rs1766 | G | A | 0.055 | 0.0097 | 1.64E-08 | 32.15007 |
| rs182695896 | C | A | 0.3852 | 0.0283 | 3.40E-42 | 185.2677 |
| rs187270432 | A | G | 0.221 | 0.0252 | 1.59E-18 | 76.91012 |
| rs217184 | C | T | -0.0872 | 0.0109 | 1.61E-15 | 64 |
| rs2497318 | T | C | 0.0548 | 0.009 | 1.14E-09 | 37.07457 |
| rs2642442 | T | C | 0.0557 | 0.0099 | 1.64E-08 | 31.65483 |
| rs2727270 | T | C | -0.1088 | 0.0101 | 5.07E-27 | 116.042 |
| rs2740486 | G | T | -0.0519 | 0.009 | 9.28E-09 | 33.25444 |
| rs3005923 | A | G | -0.4409 | 0.0288 | 8.86E-53 | 234.3663 |
| rs326 | G | A | -0.0663 | 0.0102 | 8.24E-11 | 42.25 |
| rs34931250 | T | C | 0.1052 | 0.0181 | 6.32E-09 | 33.78114 |
| rs3808348 | T | C | -0.0563 | 0.01 | 1.72E-08 | 31.6969 |
| rs41279633 | T | G | 0.0993 | 0.011 | 1.74E-19 | 81.49165 |
| rs4240624 | A | G | 0.1205 | 0.0135 | 3.94E-19 | 79.67215 |
| rs4809404 | G | A | -0.0533 | 0.0091 | 4.10E-09 | 34.30612 |
| rs4927235 | G | A | 0.0555 | 0.0091 | 1.00E-09 | 37.19659 |
| rs494198 | A | C | 0.1273 | 0.009 | 5.58E-45 | 200.0653 |
| rs507666 | A | G | 0.1445 | 0.0111 | 1.68E-38 | 169.4688 |
| rs629301 | T | G | 0.2107 | 0.0109 | 1.27E-82 | 373.6595 |
| rs6459450 | C | T | -0.0541 | 0.0092 | 4.85E-09 | 34.57951 |
| rs72831817 | T | C | -0.1288 | 0.0221 | 5.82E-09 | 33.96622 |
| rs7412 | T | C | -0.7187 | 0.0203 | 1.00E-200 | 1253.439 |
| rs7608993 | C | T | -0.0579 | 0.01 | 8.06E-09 | 33.5241 |
| rs76224003 | G | A | 0.0772 | 0.0122 | 2.66E-10 | 40.04192 |
| rs76643124 | A | G | -0.0905 | 0.0149 | 1.17E-09 | 36.89136 |
| rs76866386 | C | T | -0.2227 | 0.0162 | 5.02E-43 | 188.9776 |
| rs76895963 | G | T | -0.2241 | 0.0269 | 8.15E-17 | 69.40315 |
| rs7707394 | A | G | 0.0765 | 0.0093 | 1.29E-16 | 67.66389 |
| rs77645768 | A | G | 0.4487 | 0.028 | 6.52E-58 | 256.8006 |
| rs79915079 | A | G | -0.2609 | 0.0265 | 7.55E-23 | 96.9296 |
| rs8821 | T | C | -0.1353 | 0.0247 | 4.41E-08 | 30.00556 |
| rs9844128 | T | C | -0.0954 | 0.0164 | 6.16E-09 | 33.83834 |
| rs998584 | A | C | 0.0732 | 0.009 | 4.66E-16 | 66.15111 |

Abbreviation: SNP, single nucleotide polymorphism; EA, effect allele; OA, other allele; Beta, genetic effects of SNP on ulcerative colitis; SE, standard error

**Table S3** Genome-wide significant SNPs associated with atorvastatin use in knee osteoarthritis

| SNP | EA | OA | Beta | SE | P | F-statistic |
| --- | --- | --- | --- | --- | --- | --- |
| rs10455872 | G | A | 0.00874 | 0.000656 | 1.50E-40 | 177.715 |
| rs1169288 | C | A | 0.002501 | 0.000382 | 6.00E-11 | 42.83107 |
| rs117113213 | A | G | 0.006089 | 0.001006 | 1.40E-09 | 36.66172 |
| rs12260037 | T | C | 0.002175 | 0.000392 | 2.90E-08 | 30.75595 |
| rs12748266 | C | T | -0.00274 | 0.000436 | 3.40E-10 | 39.42095 |
| rs12916 | C | T | 0.002914 | 0.000361 | 6.50E-16 | 65.26511 |
| rs13022873 | C | A | 0.002771 | 0.000405 | 7.70E-12 | 46.83273 |
| rs17411168 | C | T | -0.00248 | 0.0004 | 6.20E-10 | 38.2556 |
| rs2569550 | C | T | 0.00255 | 0.000361 | 1.50E-12 | 50.04378 |
| rs2927472 | C | T | 0.003429 | 0.000481 | 1.00E-12 | 50.79658 |
| rs34468875 | T | C | 0.003182 | 0.000357 | 4.60E-19 | 79.60145 |
| rs429358 | C | T | 0.007119 | 0.00049 | 6.80E-48 | 211.3877 |
| rs4299376 | T | G | -0.00318 | 0.000378 | 4.00E-17 | 70.78485 |
| rs4915853 | A | C | 0.002146 | 0.00036 | 2.50E-09 | 35.54796 |
| rs58542926 | T | C | -0.00399 | 0.000673 | 3.20E-09 | 35.07901 |
| rs6093446 | A | G | 0.002243 | 0.00039 | 9.20E-09 | 33.00353 |
| rs629301 | T | G | 0.005763 | 0.000425 | 6.70E-42 | 183.9217 |

Abbreviation: SNP, single nucleotide polymorphism; EA, effect allele; OA, other allele; Beta, genetic effects of SNP on ulcerative colitis; SE, standard error

**Table S4** Genome-wide significant SNPs associated with atorvastatin use in hip osteoarthritis

| SNP | EA | OA | Beta | SE | P | F-statistic |
| --- | --- | --- | --- | --- | --- | --- |
| rs10455872 | G | A | 0.00874 | 0.000656 | 1.50E-40 | 177.715 |
| rs1169288 | C | A | 0.002501 | 0.000382 | 6.00E-11 | 42.83107 |
| rs117113213 | A | G | 0.006089 | 0.001006 | 1.40E-09 | 36.66172 |
| rs12260037 | T | C | 0.002175 | 0.000392 | 2.90E-08 | 30.75595 |
| rs12748266 | C | T | -0.00274 | 0.000436 | 3.40E-10 | 39.42095 |
| rs12916 | C | T | 0.002914 | 0.000361 | 6.50E-16 | 65.26511 |
| rs13022873 | C | A | 0.002771 | 0.000405 | 7.70E-12 | 46.83273 |
| rs17411168 | C | T | -0.00248 | 0.0004 | 6.20E-10 | 38.2556 |
| rs2519093 | T | C | 0.002543 | 0.000455 | 2.30E-08 | 31.20817 |
| rs2569550 | C | T | 0.00255 | 0.000361 | 1.50E-12 | 50.04378 |
| rs2927472 | C | T | 0.003429 | 0.000481 | 1.00E-12 | 50.79658 |
| rs34468875 | T | C | 0.003182 | 0.000357 | 4.60E-19 | 79.60145 |
| rs429358 | C | T | 0.007119 | 0.00049 | 6.80E-48 | 211.3877 |
| rs4299376 | T | G | -0.00318 | 0.000378 | 4.00E-17 | 70.78485 |
| rs4915853 | A | C | 0.002146 | 0.00036 | 2.50E-09 | 35.54796 |
| rs58542926 | T | C | -0.00399 | 0.000673 | 3.20E-09 | 35.07901 |
| rs6093446 | A | G | 0.002243 | 0.00039 | 9.20E-09 | 33.00353 |
| rs629301 | T | G | 0.005763 | 0.000425 | 6.70E-42 | 183.9217 |

Abbreviation: SNP, single nucleotide polymorphism; EA, effect allele; OA, other allele; Beta, genetic effects of SNP on ulcerative colitis; SE, standard error

**Table S5** Genome-wide significant SNPs associated with rosuvastatin use in knee osteoarthritis

| SNP | EA | OA | Beta | SE | P | F-statistic |
| --- | --- | --- | --- | --- | --- | --- |
| rs12740374 | T | G | -0.00128 | 0.000196 | 6.30E-11 | 42.72882 |
| rs17248783 | A | G | -0.00155 | 0.000247 | 3.50E-10 | 39.37458 |
| rs2954038 | A | C | -0.00117 | 0.000178 | 3.90E-11 | 43.64267 |
| rs4420638 | G | A | 0.00185 | 0.000208 | 6.80E-19 | 78.82951 |
| rs4665492 | C | A | 0.001204 | 0.000172 | 2.50E-12 | 49.08025 |

Abbreviation: SNP, single nucleotide polymorphism; EA, effect allele; OA, other allele; Beta, genetic effects of SNP on ulcerative colitis; SE, standard error

**Table S6** Genome-wide significant SNPs associated with rosuvastatin use in hip osteoarthritis

| SNP | EA | OA | Beta | SE | P | F-statistic |
| --- | --- | --- | --- | --- | --- | --- |
| rs12740374 | T | G | -0.00128 | 0.000196 | 6.30E-11 | 42.72882 |
| rs17248783 | A | G | -0.00155 | 0.000247 | 3.50E-10 | 39.37458 |
| rs2954038 | A | C | -0.00117 | 0.000178 | 3.90E-11 | 43.64267 |
| rs4420638 | G | A | 0.00185 | 0.000208 | 6.80E-19 | 78.82951 |
| rs4665492 | C | A | 0.001204 | 0.000172 | 2.50E-12 | 49.08025 |

Abbreviation: SNP, single nucleotide polymorphism; EA, effect allele; OA, other allele; Beta, genetic effects of SNP on ulcerative colitis; SE, standard error

**Table S7** Genome-wide significant SNPs associated with simvastatin use in knee osteoarthritis

| SNP | EA | OA | Beta | SE | P | F-statistic |
| --- | --- | --- | --- | --- | --- | --- |
| rs10410835 | C | T | 0.004459 | 0.000664 | 1.80E-11 | 45.14649 |
| rs1081105 | C | A | 0.013416 | 0.001998 | 1.90E-11 | 45.08535 |
| rs112552009 | G | T | -0.01516 | 0.001013 | 1.20E-50 | 224.0265 |
| rs11570891 | T | C | -0.00648 | 0.00106 | 9.60E-10 | 37.40476 |
| rs11591147 | T | G | -0.0298 | 0.002497 | 7.90E-33 | 142.4151 |
| rs11601507 | A | C | 0.007686 | 0.001271 | 1.50E-09 | 36.59232 |
| rs117733303 | G | A | 0.016057 | 0.002431 | 4.00E-11 | 43.62213 |
| rs1260326 | C | T | -0.00392 | 0.000668 | 4.50E-09 | 34.4051 |
| rs12740374 | T | G | -0.01189 | 0.000787 | 1.60E-51 | 227.9872 |
| rs12916 | C | T | 0.005008 | 0.000668 | 6.30E-14 | 56.27831 |
| rs1367117 | A | G | 0.008107 | 0.000692 | 1.10E-31 | 137.1964 |
| rs1831733 | C | T | 0.005071 | 0.000657 | 1.20E-14 | 59.52022 |
| rs2207132 | A | G | 0.013151 | 0.00182 | 5.00E-13 | 52.18642 |
| rs2618567 | T | G | -0.00407 | 0.00069 | 3.60E-09 | 34.81528 |
| rs2673134 | G | A | 0.004252 | 0.00068 | 3.90E-10 | 39.13842 |
| rs2737231 | G | A | -0.00394 | 0.000714 | 3.30E-08 | 30.49356 |
| rs2738447 | C | A | 0.005431 | 0.000667 | 3.70E-16 | 66.39515 |
| rs2980888 | C | T | -0.00783 | 0.000712 | 4.50E-28 | 120.6691 |
| rs3104415 | C | A | 0.004113 | 0.000692 | 2.80E-09 | 35.34388 |
| rs3127580 | T | C | 0.005305 | 0.000903 | 4.20E-09 | 34.54902 |
| rs35198068 | C | T | 0.005834 | 0.00072 | 5.40E-16 | 65.65286 |
| rs4299376 | T | G | -0.0068 | 0.000699 | 2.50E-22 | 94.45917 |
| rs4360309 | T | C | 0.003777 | 0.000666 | 1.40E-08 | 32.13844 |
| rs58542926 | T | C | -0.00836 | 0.001245 | 1.90E-11 | 45.05174 |
| rs693668 | A | G | 0.004127 | 0.000686 | 1.80E-09 | 36.19732 |
| rs7412 | T | C | -0.02574 | 0.001205 | 3.90E-101 | 455.8129 |
| rs7513688 | A | G | 0.00382 | 0.000684 | 2.30E-08 | 31.18809 |
| rs76895963 | G | T | -0.01442 | 0.002534 | 1.30E-08 | 32.39889 |
| rs79598313 | T | C | 0.012485 | 0.002167 | 8.40E-09 | 33.18379 |
| rs9368222 | A | C | 0.00456 | 0.000743 | 8.50E-10 | 37.63816 |

Abbreviation: SNP, single nucleotide polymorphism; EA, effect allele; OA, other allele; Beta, genetic effects of SNP on ulcerative colitis; SE, standard error

**Table S8** Genome-wide significant SNPs associated with simvastatin use in hip osteoarthritis

| SNP | EA | OA | Beta | SE | P | F-statistic |
| --- | --- | --- | --- | --- | --- | --- |
| rs10410835 | C | T | 0.004459 | 0.000664 | 1.80E-11 | 45.14649 |
| rs1081105 | C | A | 0.013416 | 0.001998 | 1.90E-11 | 45.08535 |
| rs112552009 | G | T | -0.01516 | 0.001013 | 1.20E-50 | 224.0265 |
| rs11570891 | T | C | -0.00648 | 0.00106 | 9.60E-10 | 37.40476 |
| rs11591147 | T | G | -0.0298 | 0.002497 | 7.90E-33 | 142.4151 |
| rs11601507 | A | C | 0.007686 | 0.001271 | 1.50E-09 | 36.59232 |
| rs117733303 | G | A | 0.016057 | 0.002431 | 4.00E-11 | 43.62213 |
| rs1260326 | C | T | -0.00392 | 0.000668 | 4.50E-09 | 34.4051 |
| rs12740374 | T | G | -0.01189 | 0.000787 | 1.60E-51 | 227.9872 |
| rs12916 | C | T | 0.005008 | 0.000668 | 6.30E-14 | 56.27831 |
| rs1367117 | A | G | 0.008107 | 0.000692 | 1.10E-31 | 137.1964 |
| rs1831733 | C | T | 0.005071 | 0.000657 | 1.20E-14 | 59.52022 |
| rs2207132 | A | G | 0.013151 | 0.00182 | 5.00E-13 | 52.18642 |
| rs2618567 | T | G | -0.00407 | 0.00069 | 3.60E-09 | 34.81528 |
| rs2673134 | G | A | 0.004252 | 0.00068 | 3.90E-10 | 39.13842 |
| rs2737231 | G | A | -0.00394 | 0.000714 | 3.30E-08 | 30.49356 |
| rs2738447 | C | A | 0.005431 | 0.000667 | 3.70E-16 | 66.39515 |
| rs2980888 | C | T | -0.00783 | 0.000712 | 4.50E-28 | 120.6691 |
| rs3104415 | C | A | 0.004113 | 0.000692 | 2.80E-09 | 35.34388 |
| rs3127580 | T | C | 0.005305 | 0.000903 | 4.20E-09 | 34.54902 |
| rs35198068 | C | T | 0.005834 | 0.00072 | 5.40E-16 | 65.65286 |
| rs4299376 | T | G | -0.0068 | 0.000699 | 2.50E-22 | 94.45917 |
| rs4360309 | T | C | 0.003777 | 0.000666 | 1.40E-08 | 32.13844 |
| rs58542926 | T | C | -0.00836 | 0.001245 | 1.90E-11 | 45.05174 |
| rs635634 | T | C | 0.006376 | 0.000845 | 4.50E-14 | 56.91799 |
| rs645040 | T | G | 0.004651 | 0.00078 | 2.50E-09 | 35.50772 |
| rs693668 | A | G | 0.004127 | 0.000686 | 1.80E-09 | 36.19732 |
| rs7412 | T | C | -0.02574 | 0.001205 | 3.90E-101 | 455.8129 |
| rs76895963 | G | T | -0.01442 | 0.002534 | 1.30E-08 | 32.39889 |
| rs79598313 | T | C | 0.012485 | 0.002167 | 8.40E-09 | 33.18379 |
| rs8126001 | T | C | -0.00363 | 0.000657 | 3.40E-08 | 30.49183 |
| rs9368222 | A | C | 0.00456 | 0.000743 | 8.50E-10 | 37.63816 |

Abbreviation: SNP, single nucleotide polymorphism; EA, effect allele; OA, other allele; Beta, genetic effects of SNP on ulcerative colitis; SE, standard error
